# Some new retarded nonlinear Volterra-Fredholm type integral inequalities with maxima in two variables and their applications

**DOI:** 10.1186/s13660-017-1460-6

**Published:** 2017-08-15

**Authors:** Run Xu, Xiangting Ma

**Affiliations:** Department of Mathematics, Qufu Normal University, Qufu, Shandong 273165 People’s Republic of China

**Keywords:** 42B20, 26D07, 26D15, Volterra-Fredholm type, integral inequality, integral equations, maxima, iterated integral inequality

## Abstract

In this paper, we establish some new retarded nonlinear Volterra-Fredholm type integral inequalities with maxima in two independent variables, and we present the applications to research the boundedness of solutions to retarded nonlinear Volterra-Fredholm type integral equations.

## Introduction

Gronwall-Bellman inequality [1, 2] and Bihari inequality [3] provided important devices in the study of existence, uniqueness, boundedness, oscillation, stability, invariant manifolds and other qualitative properties of solutions to differential equations, integral equations and integro-differential equations. In the past few decades, a number of studies have focused on generalizations of the Gronwall-Bellman inequality. For example, in [4–10], the Gronwall-Bellman-Gamidov type integral inequalities and their generalizations were studied; in [11–14], the Gronwall-like inequalities and their deformations were investigated; in [15–18], the Volterra type iterated inequalities were discussed; in [19–24], the Volterra-Fredholm type inequalities were examined.

The Gronwall-Bellman inequality can be stated as follows.

If *u* and *f* are nonnegative continuous functions on an interval $[a,b]$, and *u* satisfies the following inequality:

1.1 $$\begin{aligned} u(t)\leq c+ \int_{a}^{t}f(s)u(s)\,ds,\quad t\in [a,b], \end{aligned}$$

where $c\geq0$ is a constant. Then

1.2 $$\begin{aligned} u(t)\leq c\exp \biggl( \int_{a}^{t}f(s)\,ds \biggr). \end{aligned}$$

In 2004, Pachpatte [6] investigated the retarded linear Volterra-Fredholm type integral inequality in two independent variables:

1.3 $$ \begin{aligned}[b] &u(x,y)\leqslant c+ \int_{\alpha(x_{0})}^{\alpha(x)} \int_{\beta(y_{0})}^{\beta(y)}a(x,y,s,t)u(s,t)\,dt\,ds+ \int_{\alpha(x_{0})}^{\alpha(M)} \int_{\beta(y_{0})}^{\beta(N)}b(x,y,s,t)u(s,t)\,dt\,ds,\hspace{-20pt} \\ &\quad (x,y)\in [x_{0},M]\times[y_{0},N]. \end{aligned} $$

In 2010, Wang [14] investigated a retarded Volterra type integral inequality with two variables:

1.4 $$ \begin{aligned}[b] \psi \bigl(u(x,y) \bigr)&\leqslant a(x,y)+b(x,y) \int_{x_{0}}^{x} c(s,y)\psi \bigl(u(s,y) \bigr)\,ds \\ &\quad {} +d(x,y) \biggl[ \int_{\alpha_{1}(x_{0})}^{\alpha_{1}(x)} \int_{\beta_{1}(y_{0})}^{\beta_{1}(y)}f_{1}(s,t) \varphi_{1} \bigl(u(s,t) \bigr)\,dt\,ds \\ &\quad{}+ \int_{\alpha_{2}(x_{0})}^{\alpha_{2}(x)} \int_{\beta_{2}(y_{0})}^{\beta_{2}(y)}f_{2}(s,t) \varphi_{2} \bigl(u(s,t) \bigr)\,dt\,ds \biggr],\quad (x,y)\in [x_{0},x_{1})\times[y_{0},y_{1}). \end{aligned} $$

In 2014, Lu *et al.* [21] studied the nonlinear retarded Volterra-Fredholm type iterated integral inequality:

1.5 $$ \begin{aligned}[b] u(x,y) &\leqslant k+ \int_{\alpha(x_{0})}^{\alpha(x)} \int_{\beta(y_{0})}^{\beta(y)}h_{1}(s_{1},t_{1}) \omega \bigl(u(s_{1},t_{1}) \bigr) \\ &\quad {} \times \biggl[f_{1}(s_{1},t_{1}) \omega_{1} \bigl(u(s_{1},t_{1}) \bigr)+ \int_{\alpha(x_{0})}^{s_{1}} \int_{\beta(y_{0})}^{t_{1}}h_{2}(s_{2},t_{2}) \biggl[f_{2}(s_{2},t_{2}) \omega_{2} \bigl(u(s_{2},t_{2}) \bigr) \\ &\quad {}+ \int_{\alpha(x_{0})}^{s_{2}} \int_{\beta(y_{0})}^{t_{2}} h_{3}(s_{3},t_{3}) \omega_{3} \bigl(u(s_{3},t_{3}) \bigr) \,dt_{3}\,ds_{3} \biggr]\,dt_{2}\,ds_{2} \biggr]\,dt_{1}\,ds_{1} \\ &\quad{}+ \int_{\alpha(x_{0})}^{\alpha(M)} \int_{\beta(y_{0})}^{\beta(N)}h_{1}(s_{1},t_{1}) \omega \bigl(u(s_{1},t_{1}) \bigr) \biggl[f_{1}(s_{1},t_{1}) \omega_{1} \bigl(u(s_{1},t_{1}) \bigr) \\ &\quad {}+ \int_{\alpha(x_{0})}^{s_{1}} \int_{\beta(y_{0})}^{t_{1}}h_{2}(s_{2},t_{2}) \biggl[f_{2}(s_{2},t_{2}) \omega_{2} \bigl(u(s_{2},t_{2}) \bigr) \\ &\quad{}+ \int_{\alpha(x_{0})}^{s_{2}} \int_{\beta(y_{0})}^{t_{2}} h_{3}(s_{3},t_{3}) \omega_{3} \bigl(u(s_{3},t_{3}) \bigr) \,dt_{3}\,ds_{3} \biggr]\,dt_{2}\,ds_{2} \biggr]\,dt_{1}\,ds_{1},\\ & \quad (x,y)\in \bigtriangleup. \end{aligned} $$

In 2016, Huang and Wang [23] discussed the retarded nonlinear Volterra-Fredholm type integral inequality with maxima:

1.6 $$ \begin{gathered} \varphi \bigl(v(t) \bigr)\leq k+ \int_{\alpha(t_{0})}^{\alpha(t)}h_{1}(s) \biggl[f_{1}(s)\phi_{1} \bigl(v(s) \bigr)+ \int_{\alpha(t_{0})}^{s}h_{2}(\tau) \biggl[f_{2}(\tau)\phi_{2} \bigl(v(\tau) \bigr) \\ \hphantom{\varphi (v(t) )\leq}{}+ \int_{\alpha(t_{0})}^{\tau}h_{3}(\xi) \phi_{3} \Bigl(\max_{\eta\in[\xi-h,\xi]}v(\eta) \Bigr)\,d\xi \biggr] \,d \tau \biggr]\,ds \\ \hphantom{\varphi (v(t) )\leq}{}+ \int_{\alpha(t_{0})}^{\alpha(T)}h_{1}(s) \biggl[f_{1}(s)\phi_{1} \bigl(v(s) \bigr)+ \int_{\alpha(t_{0})}^{s}h_{2}(\tau) \biggl[f_{2}(\tau)\phi_{2} \bigl(v(\tau) \bigr) \\ \hphantom{\varphi (v(t) )\leq}{}+ \int_{\alpha(t_{0})}^{\tau}h_{3}(\xi) \phi_{3} \Bigl(\max_{\eta\in[\xi-h,\xi]}v(\eta) \Bigr)\,d\xi \biggr] \,d \tau \biggr]\,ds,\quad t\in[t_{0},T], \\ v(t)\leq k,\quad t\in[t_{0}-h,t_{0}]. \end{gathered} $$

Motivated by the work presented in [14, 21, 23], we establish some new retarded nonlinear Volterra-Fredholm type integral inequality with maxima in two independent variables in this paper:

1.7 $$\begin{aligned} \psi \bigl(u(x,y) \bigr)&\leq k(x,y)+ \int_{\alpha(x)}^{\infty}a(s,y)\psi \bigl(u(s,y) \bigr)\,ds +\sum _{i=1}^{l_{1}} \int_{\alpha_{i}(x)}^{\infty} \int_{\beta_{i}(y)}^{\infty} \biggl[b_{i}(s,t,x,y) \varphi_{1} \bigl(u(s,t) \bigr) \\ &\quad{}+ \int_{s}^{\infty} \int_{t}^{\infty}c_{i}(\xi,\eta,x,y) \varphi_{2} \Bigl(\max_{\sigma\in [\xi,h\xi]} u(\sigma,\eta) \Bigr)\,d \xi \,d \eta \biggr]\,ds\,dt \\ &\quad {} +\sum_{j=1}^{l_{2}} \int_{\alpha_{j}(M)}^{\infty} \int_{\beta_{j}(N)}^{\infty} \biggl[d_{j}(s,t,x,y)\psi \bigl(u(s,t) \bigr) \\ &\quad{}+ \int_{s}^{\infty} \int_{t}^{\infty}e_{j}(\xi,\eta,x,y)\psi \Bigl( \max_{\sigma\in [\xi,h\xi]} u(\sigma,\eta) \Bigr)\,d\xi \,d\eta \biggr] \,ds\,dt,\quad (x,y)\in\Delta, \end{aligned}$$

and

1.8 $$\begin{aligned} u^{p}(x,y)&\leq k(x,y)+ \int_{\alpha(x)}^{\infty}a(s,y)u^{p}(s,y)\,ds +\sum _{i=1}^{l_{1}} \int_{\alpha_{i}(x)}^{\infty} \int_{\beta_{i}(y)}^{\infty} \biggl[b_{i}(s,t,x,y)u^{q_{i}}(s,t) \\ &\quad{}+ \int_{s}^{\infty} \int_{t}^{\infty}c_{i}(\xi,\eta,x,y)\max _{\sigma\in [\xi,h\xi]} u^{r_{i}}(\sigma,\eta)\,d\xi \,d\eta \biggr]\,ds \,dt \\ &\quad {} +\sum_{j=1}^{l_{2}} \int_{\alpha_{j}(M)}^{\infty} \int_{\beta_{j}(N)}^{\infty} \biggl[d_{j}(s,t,x,y)u^{\varepsilon_{j}}(s,t) \\ &\quad{}+ \int_{s}^{\infty} \int_{t}^{\infty}e_{j}(\xi,\eta,x,y)\max _{\sigma\in [\xi,h\xi]} u^{\delta_{j}}(\sigma,\eta)\,d\xi \,d\eta \biggr]\,ds \,dt, \quad (x,y)\in\Delta. \end{aligned}$$

By the amplification method, differential and integration, and the inverse function, we obtain the lower bound estimation of the unknown function. The example is given to illustrate the application of our results.

## Main results

In what follows, *R* denotes the set of real numbers, $R_{+}=[0,+\infty)$, $I_{1}=[M,+\infty)$, $I_{2}=[N,+\infty)$ are the given subsets of *R*, $\Delta=I_{1}\times I_{2} $. $C^{1}(\Omega,S)$ denotes the class of all continuously differentiable functions defined on set Ω with range in the set *S*, $C(\Omega,S)$ denotes the class of all continuous functions defined on set Ω with range in the set *S*, and $\alpha'(t)$ denotes the derived function of a function $\alpha(t)$. For convenience, we cite some useful lemmas in the discussion of our proof as follows:

### Lemma 2.1

See [25]

*Let* $u(t)$, $a(t)$, $b(t)$ *be nonnegative and continuous functions defined for* $t\in R_{+}$. *Assume that* $a(t)$ *is non*-*increasing function for* $t\in R_{+}$. *If*

$$u(t)\leq a(t)+ \int_{t}^{\infty}b(s)u(s)\,ds,\quad t\in R_{+}, $$

*then*

$$u(t)\leq a(t)\exp{ \biggl( \int_{t}^{\infty}b(s)\,ds \biggr)},\quad t\in R_{+}. $$

From Lemma 2.1, we can get the generalization in two dimensions.

### Lemma 2.2

*Let* $u(x,y)$, $a(x,y)$, $b(x,y)$ *be nonnegative and continuous functions defined for* $(x,y)\in\Delta$. *Assume that* $a(x,y)$ *is a non*-*increasing function in the first variable*. *If*

$$\begin{aligned} u(x,y)\leq a(x,y)+ \int_{x}^{\infty}b(s,y)u(s,y)\,ds,\quad (x,y)\in\Delta, \end{aligned}$$

*then*

$$\begin{aligned} u(x,y)\leq a(x,y)\exp \biggl( \int_{x}^{\infty}b(s,y)\,ds \biggr),\quad (x,y)\in \Delta. \end{aligned}$$

### Lemma 2.3

See [26]

*Assume that* $a\geq0$, $p\geq q\geq 0$, *and* $p\neq0$. *Then*, *for any* $K>0$,

$$\begin{aligned} a^{\frac{q}{p}}\leq{\frac{q}{p}}K^{\frac{q-p}{p}}a+{\frac{p-q}{p}}K^{\frac{q}{p}}. \end{aligned}$$

### Theorem 2.1

*Suppose that the following conditions hold*:

(i) $\psi\in C(R_{+},R_{+})$ *is an increasing function and* $\psi(u)>0$, $\forall u>0$, $\psi(\infty)=\infty$. $\psi^{-1}$ *is the inverse function of* *ψ*. $\varphi_{1}$, $\varphi_{2}$, $\varphi_{2}/\varphi_{1}\in C(R_{+},R_{+})$ *are increasing functions with* $\varphi_{i}(u)>0$ ($i=1,2$) *for* $u>0$. $\psi^{-1}$, $\varphi_{i}$ ($i=1,2$) *are sub*-*multiplicative and sub*-*additive*, *that is*,
  $$\begin{gathered} \psi^{-1}(\alpha\beta)\leq \psi^{-1}( \alpha)\psi^{-1}(\beta),\qquad \psi^{-1}(\alpha+\beta)\leq \psi^{-1}(\alpha)+\psi^{-1}(\beta), \\ \varphi_{i}(\alpha\beta)\leq \varphi_{i}(\alpha) \varphi_{i}(\beta),\qquad \varphi_{i}(\alpha+\beta)\leq \varphi_{i}(\alpha)+\varphi_{i}(\beta), \quad \alpha,\beta \in R_{+}; \end{gathered} $$
(ii) $k(x,y), a(x,y)\in C(\Delta,R_{+})$ *and* $k(x,y)$ *is non*-*increasing in the first variable*;
(iii) $b_{i}(s,t,x,y), c_{i}(s,t,x,y), d_{j}(s,t,x,y), e_{j}(s,t,x,y)\in C(\Delta^{2},R_{+})$ *for* $i=1,2,\ldots,l_{1}$; $j=1,2,\ldots,l_{2}$; $b_{i}$, $c_{i}$, $d_{j}$, $e_{j}$ *are all non*-*increasing functions in the last two variables*;
(iv) $\alpha, \alpha_{i}, \alpha_{j}\in C(I_{1},I_{1})$, $\beta_{i}, \beta_{j}\in C(I_{2},I_{2})$ *are non*-*decreasing functions with* $\alpha(x), \alpha_{i}(x), \alpha_{j}(x)\geq x$, $\beta_{i}(y), \beta_{j}(y)\geq y$ ($i=1,2,\ldots,l_{1}$; $j=1,2,\ldots,l_{2}$). $h\geq1$ *is a constant*;
(v) *the function* $u\in C(\Delta,R_{+})$ *satisfies the inequality*
  2.1 $$\begin{aligned} \psi \bigl(u(x,y) \bigr)&\leq k(x,y)+ \int_{\alpha(x)}^{\infty}a(s,y)\psi \bigl(u(s,y) \bigr)\,ds \\ &\quad {} +\sum _{i=1}^{l_{1}} \int_{\alpha_{i}(x)}^{\infty} \int_{\beta_{i}(y)}^{\infty} \biggl[b_{i}(s,t,x,y) \varphi_{1} \bigl(u(s,t) \bigr) \\ &\quad{}+ \int_{s}^{\infty} \int_{t}^{\infty}c_{i}(\xi,\eta,x,y) \varphi_{2} \Bigl(\max_{\sigma\in [\xi,h\xi]} u(\sigma,\eta) \Bigr)\,d \xi \,d \eta \biggr]\,ds\,dt \\ &\quad {} +\sum_{j=1}^{l_{2}} \int_{\alpha_{j}(M)}^{\infty} \int_{\beta_{j}(N)}^{\infty} \biggl[d_{j}(s,t,x,y)\psi \bigl(u(s,t) \bigr) \\ &\quad {}+ \int_{s}^{\infty} \int_{t}^{\infty}e_{j}(\xi,\eta,x,y)\psi \Bigl( \max_{\sigma\in [\xi,h\xi]} u(\sigma,\eta) \Bigr)\,d\xi \,d\eta \biggr] \,ds\,dt, \\ &\quad (x,y)\in\Delta, \end{aligned}$$

*then we have*

2.2 $$\begin{aligned} u(x,y)&\leq \psi^{-1} \bigl\{ \bigl[k(x,y)+W_{1}^{-1} \bigl\{ W_{2}^{-1} \bigl\{ W_{2} \bigl[W_{1} \bigl(B(M,N)+G^{-1} \bigl(F(M,N) \bigr) \bigr) \\ &\quad {}+E(x,y) \bigr]+F(x,y) \bigr\} \bigr\} \bigr]A(x,y) \bigr\} , \end{aligned}$$

*where*

2.3 $$\begin{aligned} &W_{1}(z)= \int_{c}^{z}\frac{ds}{\varphi_{1}(\psi^{-1}(s))},\quad c>0, z\in (0,+ \infty), \end{aligned}$$

2.4 $$\begin{aligned} &W_{2}(z)= \int_{c}^{z}\frac{\varphi_{1}(\psi^{-1}(W_{1}^{-1}(s)))}{\varphi_{2}(\psi^{-1}(W_{1}^{-1}(s)))}\,ds,\quad c>0, z\in (0,+\infty), \end{aligned}$$

2.5 $$\begin{aligned} &G(u)=W_{2} \biggl(W_{1} \biggl(\frac{u}{D(M,N)} \biggr) \biggr)-W_{2} \bigl(W_{1} \bigl(B(M,N)+u \bigr)+E(M,N) \bigr), \end{aligned}$$

*on condition that* $W_{1}(+\infty)=+\infty$, $W_{2}(+\infty)=+\infty$, *and* $G(u)$ *is a strictly increasing function on* $R_{+}$. *We have*

2.6 $$\begin{aligned}& A(x,y)= \exp \biggl( \int_{\alpha(x)}^{\infty}a(s,y)\,ds \biggr), \end{aligned}$$

2.7 $$\begin{aligned}& \begin{aligned}[b] B(M,N)&= \sum_{i=1}^{l_{1}} \int_{\alpha_{i}(M)}^{\infty} \int_{\beta_{i}(N)}^{\infty} \biggl[b_{i}(s,t,M,N) \varphi_{1} \bigl(\psi^{-1} \bigl(k(s,t)A(s,t) \bigr) \bigr) \\ &\quad {}+ \int_{s}^{\infty} \int_{t}^{\infty}c_{i}(\xi,\eta,M,N) \varphi_{2} \bigl(\psi^{-1} \bigl(k(\xi,\eta)A(\xi,\eta) \bigr) \bigr)\,d\xi \,d\eta \biggr]\,ds\,dt \\ &\quad {}+\sum_{j=1}^{l_{2}} \int_{\alpha_{j}(M)}^{\infty} \int_{\beta_{j}(N)}^{\infty} \biggl[d_{j}(s,t,M,N)k(s,t)A(s,t) \\ &\quad {}+ \int_{s}^{\infty} \int_{t}^{\infty}e_{j}(\xi,\eta,M,N)k(\xi, \eta)A(\xi,\eta)\,d\xi \,d\eta \biggr]\,ds\,dt, \end{aligned} \end{aligned}$$

2.8 $$\begin{aligned}& \begin{aligned}[b] D(M,N)&= \sum_{j=1}^{l_{2}} \int_{\alpha_{j}(M)}^{\infty} \int_{\beta_{j}(N)}^{\infty} \biggl[d_{j}(s,t,M,N)A(s,t)\\ &\quad {}+ \int_{s}^{\infty} \int_{t}^{\infty}e_{j}(\xi,\eta,M,N)A(\xi, \eta)\,d\xi \,d\eta \biggr]\,ds\,dt, \end{aligned} \end{aligned}$$

2.9 $$\begin{aligned}& E(M,N)= \sum_{i=1}^{l_{1}} \int_{\alpha_{i}(M)}^{\infty} \int_{\beta_{i}(N)}^{\infty} b_{i}(s,t,M,N) \varphi_{1} \bigl(\psi^{-1} \bigl(A(s,t) \bigr) \bigr)\,ds \,dt, \end{aligned}$$

2.10 $$\begin{aligned}& F(x,y)= \sum_{i=1}^{l_{1}} \int_{\alpha_{i}(x)}^{\infty} \int_{\beta_{i}(y)}^{\infty} \biggl[ \int_{s}^{\infty} \int_{t}^{\infty}c_{i}(\xi,\eta,x,y) \varphi_{2} \bigl(\psi^{-1} \bigl(A(\xi,\eta) \bigr) \bigr) \,d\xi \,d\eta \biggr]\,ds\,dt. \end{aligned}$$

### Proof

Let

2.11 $$\begin{aligned} z(x,y)&=\sum_{i=1}^{l_{1}} \int_{\alpha_{i}(x)}^{\infty} \int_{\beta_{i}(y)}^{\infty} \biggl[b_{i}(s,t,x,y) \varphi_{1} \bigl(u(s,t) \bigr) \\ &\quad {}+ \int_{s}^{\infty} \int_{t}^{\infty}c_{i}(\xi,\eta,x,y) \varphi_{2} \Bigl(\max_{\sigma\in [\xi,h\xi]} u(\sigma,\eta) \Bigr)\,d \xi \,d \eta \biggr]\,ds\,dt \\ &\quad {}+\sum_{j=1}^{l_{2}} \int_{\alpha_{j}(M)}^{\infty} \int_{\beta_{j}(N)}^{\infty} \biggl[d_{j}(s,t,x,y)\psi \bigl(u(s,t) \bigr) \\ &\quad{}+ \int_{s}^{\infty} \int_{t}^{\infty}e_{j}(\xi,\eta,x,y)\psi \Bigl( \max_{\sigma\in [\xi,h\xi]} u(\sigma,\eta) \Bigr)\,d\xi \,d\eta \biggr] \,ds\,dt. \end{aligned}$$

Obviously, $z(x,y)$ is non-increasing in each of the variables. From (2.1), we have

2.12 $$ \psi \bigl(u(x,y) \bigr)\leq k(x,y)+z(x,y)+ \int_{\alpha(x)}^{\infty}a(s,y)\psi \bigl(u(s,y) \bigr)\,ds. $$

Applying Lemma 2.2, we obtain

2.13 $$ \psi \bigl(u(x,y) \bigr)\leq \bigl(k(x,y)+z(x,y) \bigr)A(x,y), $$

*i.e.*

2.14 $$ u(x,y)\leq \psi^{-1} \bigl[ \bigl(k(x,y)+z(x,y) \bigr)A(x,y) \bigr], $$

where $A(x,y)$ is defined in (2.6), and $A(x,y)$ is non-increasing in the first variable. So we have

2.15 $$\begin{aligned} \max_{\xi\in[x,hx]}u(\xi,y)&\leq \max_{\xi\in[x,hx]} \psi^{-1} \bigl[ \bigl(k(\xi,y)+z(\xi,y) \bigr)A(\xi,y) \bigr] \\ &\leq \psi^{-1} \Bigl[ \max_{\xi\in[x,hx]} \bigl(k(\xi,y)A( \xi,y) \bigr)+ \max_{\xi\in[x,hx]} \bigl(A(\xi,y)z(\xi,y) \bigr) \Bigr] \\ &\leq \psi^{-1} \bigl[ k(x,y)A(x,y)+A(x,y)z(x,y) \bigr]. \end{aligned}$$

By (2.11), (2.14), (2.15), and condition (i), we deduce

2.16 $$\begin{aligned} z(x,y)&\leq \sum_{i=1}^{l_{1}} \int_{\alpha_{i}(x)}^{\infty} \int_{\beta_{i}(y)}^{\infty} \biggl[b_{i}(s,t,x,y) \varphi_{1} \bigl(\psi^{-1} \bigl( k(s,t)A(s,t) \bigr)+ \psi^{-1} \bigl(A(s,t) \bigr)\psi^{-1} \bigl(z(s,t) \bigr) \bigr) \\ &\quad{}+ \int_{s}^{\infty} \int_{t}^{\infty}c_{i}(\xi,\eta,x,y) \varphi_{2} \bigl(\psi^{-1} \bigl( k(\xi,\eta)A(\xi,\eta) \bigr) \\ &\quad {} +\psi^{-1} \bigl(A(\xi,\eta) \bigr)\psi^{-1} \bigl(z( \xi,\eta) \bigr) \bigr)\,d\xi \,d\eta \biggr]\,ds\,dt \\ &\quad{}+\sum_{j=1}^{l_{2}} \int_{\alpha_{j}(M)}^{\infty} \int_{\beta_{j}(N)}^{\infty} \biggl[d_{j}(s,t,x,y) \bigl(k(s,t)A(s,t)+A(s,t)z(s,t) \bigr) \\ &\quad{}+ \int_{s}^{\infty} \int_{t}^{\infty}e_{j}(\xi,\eta,x,y) \bigl(k( \xi,\eta)A(\xi,\eta)+A(\xi,\eta)z(\xi,\eta) \bigr)\,d\xi \,d\eta \biggr]\,ds\,dt \\ &\leq \sum_{i=1}^{l_{1}} \int_{\alpha_{i}(x)}^{\infty} \int_{\beta_{i}(y)}^{\infty} \biggl\{ b_{i}(s,t,x,y) \bigl[\varphi_{1} \bigl(\psi^{-1} \bigl( k(s,t)A(s,t) \bigr) \bigr) \\ &\quad{}+\varphi_{1} \bigl(\psi^{-1} \bigl(A(s,t) \bigr) \bigr) \varphi_{1} \bigl(\psi^{-1} \bigl(z(s,t) \bigr) \bigr) \bigr] \\ &\quad{}+ \int_{s}^{\infty} \int_{t}^{\infty}c_{i}(\xi,\eta,x,y) \bigl[ \varphi_{2} \bigl(\psi^{-1} \bigl( k(\xi,\eta)A(\xi,\eta) \bigr) \bigr) \\ &\quad{}+\varphi_{2} \bigl(\psi^{-1} \bigl(A(\xi,\eta) \bigr) \bigr)\varphi_{2} \bigl(\psi^{-1} \bigl(z(\xi,\eta) \bigr) \bigr) \bigr]\,d\xi \,d\eta \biggr\} \,ds\,dt \\ &\quad{}+\sum_{j=1}^{l_{2}} \int_{\alpha_{j}(M)}^{\infty} \int_{\beta_{j}(N)}^{\infty} \biggl\{ d_{j}(s,t,x,y) \bigl[k(s,t)A(s,t)+A(s,t)z(s,t) \bigr] \\ &\quad{}+ \int_{s}^{\infty} \int_{t}^{\infty}e_{j}(\xi,\eta,x,y) \bigl[k( \xi,\eta)A(\xi,\eta)+A(\xi,\eta)z(\xi,\eta) \bigr]\,d\xi \,d\eta \biggr\} \,ds\,dt \\ &\leq B(x,y)+C(x,y) \\ &\quad{}+\sum_{i=1}^{l_{1}} \int_{\alpha_{i}(x)}^{\infty} \int_{\beta_{i}(y)}^{\infty} \biggl[b_{i}(s,t,x,y) \varphi_{1} \bigl(\psi^{-1} \bigl(A(s,t) \bigr) \bigr) \varphi_{1} \bigl(\psi^{-1} \bigl(z(s,t) \bigr) \bigr) \\ &\quad{}+ \int_{s}^{\infty} \int_{t}^{\infty}c_{i}(\xi,\eta,x,y) \varphi_{2} \bigl(\psi^{-1} \bigl(A(\xi,\eta) \bigr) \bigr) \varphi_{2} \bigl(\psi^{-1} \bigl(z(\xi,\eta) \bigr) \bigr)\,d \xi \,d \eta \biggr]\,ds\,dt \\ &\leq B(M,N)+C(M,N) \\ &\quad {}+\sum_{i=1}^{l_{1}} \int_{\alpha_{i}(x)}^{\infty} \int_{\beta_{i}(y)}^{\infty} \biggl[b_{i}(s,t,x,y) \varphi_{1} \bigl(\psi^{-1} \bigl(A(s,t) \bigr) \bigr) \varphi_{1} \bigl(\psi^{-1} \bigl(z(s,t) \bigr) \bigr) \\ &\quad{}+ \int_{s}^{\infty} \int_{t}^{\infty}c_{i}(\xi,\eta,x,y) \varphi_{2} \bigl(\psi^{-1} \bigl(A(\xi,\eta) \bigr) \bigr) \varphi_{2} \bigl(\psi^{-1} \bigl(z(\xi,\eta) \bigr) \bigr)\,d \xi \,d \eta \biggr]\,ds\,dt, \\ &\quad \forall(x,y)\in[X,\infty)\times[Y,\infty), \end{aligned}$$

where $B(M,N)$ is defined in (2.7), and $C(M,N)$ is defined as follows:

2.17 $$\begin{aligned} C(M,N)&=\sum_{j=1}^{l_{2}} \int_{\alpha_{j}(M)}^{\infty} \int_{\beta_{j}(N)}^{\infty} \biggl[d_{j}(s,t,M,N)A(s,t)z(s,t) \\ &\quad{}+ \int_{s}^{\infty} \int_{t}^{\infty}e_{j}(\xi,\eta,M,N)A(\xi, \eta)z(\xi,\eta)\,d\xi \,d\eta \biggr]\,ds\,dt. \end{aligned}$$

$\forall X\in I_{1}$, $Y\in I_{2}$, for all $(x,y)\in[X,\infty)\times[Y,\infty)$, we have

2.18 $$\begin{aligned} z(x,y)&\leq B(M,N)+C(M,N) \\ &\quad {}+\sum_{i=1}^{l_{1}} \int_{\alpha_{i}(x)}^{\infty} \int_{\beta_{i}(y)}^{\infty} \biggl[b_{i}(s,t,X,Y) \varphi_{1} \bigl(\psi^{-1} \bigl(A(s,t) \bigr) \bigr) \varphi_{1} \bigl(\psi^{-1} \bigl(z(s,t) \bigr) \bigr) \\ &\quad{}+ \int_{s}^{\infty} \int_{t}^{\infty}c_{i}(\xi,\eta,X,Y) \varphi_{2} \bigl(\psi^{-1} \bigl(A(\xi,\eta) \bigr) \bigr) \varphi_{2} \bigl(\psi^{-1} \bigl(z(\xi,\eta) \bigr) \bigr)\,d \xi \,d \eta \biggr]\,ds\,dt.\hspace{-20pt} \end{aligned}$$

Let $z_{1}(x,y)$ denote the function on the right-hand side of (2.18), which is positive and non-increasing in each of the variables $(x,y)\in[X,\infty)\times[Y,\infty)$. From (2.18), we have

2.19 $$\begin{aligned} &z(x,y)\leq z_{1}(x,y),\quad \forall(x,y)\in[X,\infty)\times[Y, \infty), \end{aligned}$$

2.20 $$\begin{aligned} &z_{1}(\infty,y)=B(M,N)+C(M,N). \end{aligned}$$

Differentiating $z_{1}(x,y)$ with respect to *x*, we have

2.21 $$ \begin{aligned}[b] \frac{\partial z_{1}(x,y)}{\partial x}&=-\sum_{i=1}^{l_{1}} \alpha_{i}'(x) \int_{\beta_{i}(y)}^{\infty} \biggl[b_{i} \bigl( \alpha_{i}(x),t,X,Y \bigr)\varphi_{1} \bigl( \psi^{-1} \bigl(A \bigl(\alpha_{i}(x),t \bigr) \bigr) \bigr) \varphi_{1} \bigl(\psi^{-1} \bigl(z \bigl( \alpha_{i}(x),t \bigr) \bigr) \bigr)\hspace{-30pt} \\ &\quad{}+ \int_{\alpha_{i}(x)}^{\infty} \int_{t}^{\infty}c_{i}(\xi,\eta,X,Y) \varphi_{2} \bigl(\psi^{-1} \bigl(A(\xi,\eta) \bigr) \bigr) \varphi_{2} \bigl(\psi^{-1} \bigl(z(\xi,\eta) \bigr) \bigr)\,d \xi \,d \eta \biggr]\,dt\hspace{-30pt} \\ &\geq-\sum_{i=1}^{l_{1}} \alpha_{i}'(x) \int_{\beta_{i}(y)}^{\infty} \biggl[b_{i} \bigl( \alpha_{i}(x),t,X,Y \bigr)\varphi_{1} \bigl( \psi^{-1} \bigl(A \bigl(\alpha_{i}(x),t \bigr) \bigr) \bigr) \varphi_{1} \bigl(\psi^{-1} \bigl(z_{1} \bigl( \alpha_{i}(x),t \bigr) \bigr) \bigr)\hspace{-30pt} \\ &\quad{}+ \int_{\alpha_{i}(x)}^{\infty} \int_{t}^{\infty}c_{i}(\xi,\eta,X,Y) \varphi_{2} \bigl(\psi^{-1} \bigl(A(\xi,\eta) \bigr) \bigr) \varphi_{2} \bigl(\psi^{-1} \bigl(z_{1}(\xi,\eta) \bigr) \bigr)\,d\xi \,d\eta \biggr]\,dt,\hspace{-30pt} \\ &\quad\forall(x,y)\in[X,\infty)\times[Y,\infty). \end{aligned} $$

By the monotonicity of $\varphi_{1}$, $\varphi_{2}$, $z_{1}$ and the property of $\alpha_{i}$, $\beta_{i}$, from (2.21), we get

2.22 $$ \begin{aligned}[b] &\frac{(\partial/\partial x)z_{1}(x,y)}{\varphi_{1}(\psi^{-1}(z_{1}(x,y)))}\\ &\quad \geq -\sum_{i=1}^{l_{1}} \alpha_{i}'(x) \int_{\beta_{i}(y)}^{\infty} \biggl[b_{i} \bigl( \alpha_{i}(x),t,X,Y \bigr)\varphi_{1} \bigl( \psi^{-1} \bigl(A \bigl(\alpha_{i}(x),t \bigr) \bigr) \bigr) \\ &\qquad{}+ \int_{\alpha_{i}(x)}^{\infty} \int_{t}^{\infty}c_{i}(\xi,\eta,X,Y) \varphi_{2} \bigl(\psi^{-1} \bigl(A(\xi,\eta) \bigr) \bigr) \frac{\varphi_{2}(\psi^{-1}(z_{1}(\xi,\eta)))}{\varphi_{1}(\psi^{-1}(z_{1}(\xi,\eta)))}\,d\xi \,d\eta \biggr]\,dt, \\ &\qquad\forall(x,y)\in[X,\infty)\times[Y,\infty). \end{aligned} $$

Replacing *x* with *s*, and integrating it from *x* to ∞, we obtain

2.23 $$\begin{aligned} &W_{1} \bigl(z_{1}(\infty,y) \bigr)-W_{1} \bigl(z_{1}(x,y) \bigr) \\ &\quad\geq -\sum_{i=1}^{l_{1}} \int_{\alpha_{i}(x)}^{\infty} \int_{\beta_{i}(y)}^{\infty} \biggl[b_{i}(s,t,X,Y) \varphi_{1} \bigl(\psi^{-1} \bigl(A(s,t) \bigr) \bigr) \\ &\qquad{}+ \int_{s}^{\infty} \int_{t}^{\infty}c_{i}(\xi,\eta,X,Y) \varphi_{2} \bigl(\psi^{-1} \bigl(A(\xi,\eta) \bigr) \bigr) \\ &\qquad {}\times\frac{\varphi_{2}(\psi^{-1}(z_{1}(\xi,\eta)))}{\varphi_{1}(\psi^{-1}(z_{1}(\xi,\eta)))}\,d\xi \,d\eta \biggr]\,ds\,dt, \quad \forall(x,y)\in[X,\infty) \times[Y,\infty), \end{aligned}$$

*i.e.*

2.24 $$\begin{aligned} W_{1} \bigl(z_{1}(x,y) \bigr)&\leq W_{1} \bigl(z_{1}(\infty,y) \bigr)+\sum_{i=1}^{l_{1}} \int_{\alpha_{i}(x)}^{\infty} \int_{\beta_{i}(y)}^{\infty} \biggl[b_{i}(s,t,X,Y) \varphi_{1} \bigl(\psi^{-1} \bigl(A(s,t) \bigr) \bigr) \\ &\quad{}+ \int_{s}^{\infty} \int_{t}^{\infty}c_{i}(\xi,\eta,X,Y) \varphi_{2} \bigl(\psi^{-1} \bigl(A(\xi,\eta) \bigr) \bigr) \frac{\varphi_{2}(\psi^{-1}(z_{1}(\xi,\eta)))}{\varphi_{1}(\psi^{-1}(z_{1}(\xi,\eta)))}\,d\xi \,d\eta \biggr]\,ds\,dt \\ &\leq W_{1} \bigl(z_{1}(\infty,y) \bigr)+E(X,Y)+\sum _{i=1}^{l_{1}} \int_{\alpha_{i}(x)}^{\infty} \int_{\beta_{i}(y)}^{\infty} \biggl[ \int_{s}^{\infty} \int_{t}^{\infty}c_{i}(\xi,\eta,X,Y) \\ &\quad{}\times\varphi_{2} \bigl(\psi^{-1} \bigl(A(\xi,\eta) \bigr) \bigr)\frac{\varphi_{2}(\psi^{-1}(z_{1}(\xi,\eta)))}{\varphi_{1}(\psi^{-1}(z_{1}(\xi,\eta)))}\,d\xi \,d\eta \biggr]\,ds\,dt, \end{aligned}$$

where $E(X,Y)$ is defined in (2.9). Let $z_{2}(x,y)$ denote the function on the right-hand side of (2.24), which is positive and non-increasing in each of the variables $(x,y)\in[X,\infty)\times[Y,\infty)$. From (2.24), we have

2.25 $$\begin{aligned} &z_{1}(x,y)\leq W_{1}^{-1} \bigl(z_{2}(x,y) \bigr), \quad\forall(x,y)\in[X,\infty)\times[Y,\infty), \end{aligned}$$

2.26 $$\begin{aligned} &z_{2}(\infty,y)=W_{1} \bigl(z_{1}(\infty,y) \bigr)+E(X,Y). \end{aligned}$$

Differentiating $z_{2}(x,y)$ with respect to *x*, we have

2.27 $$\begin{aligned} \frac{\partial z_{2}(x,y)}{\partial x}&=-\sum_{i=1}^{l_{1}} \alpha_{i}'(x) \int_{\beta_{i}(y)}^{\infty} \biggl[ \int_{\alpha_{i}(x)}^{\infty} \int_{t}^{\infty}c_{i}(\xi,\eta,X,Y) \varphi_{2} \bigl(\psi^{-1} \bigl(A(\xi,\eta) \bigr) \bigr) \\ &\quad{}\times \frac{\varphi_{2}(\psi^{-1}(z_{1}(\xi,\eta)))}{\varphi_{1}(\psi^{-1}(z_{1}(\xi,\eta)))}\,d\xi \,d\eta \biggr]\,dt \\ &\geq -\sum_{i=1}^{l_{1}} \alpha_{i}'(x) \int_{\beta_{i}(y)}^{\infty} \biggl[ \int_{\alpha_{i}(x)}^{\infty} \int_{t}^{\infty}c_{i}(\xi,\eta,X,Y) \varphi_{2} \bigl(\psi^{-1} \bigl(A(\xi,\eta) \bigr) \bigr) \\ & \quad{}\times\frac{\varphi_{2}(\psi^{-1}(W_{1}^{-1}(z_{2}(\xi,\eta))))}{\varphi_{1}(\psi^{-1}(W_{1}^{-1}(z_{2}(\xi,\eta))))}\,d\xi \,d\eta \biggr]\,dt. \end{aligned}$$

By the monotonicity of $\varphi_{2}/\varphi_{1}$ and $z_{2}$, from (2.27), we obtain

2.28 $$\begin{aligned} &\frac{\varphi_{1}(\psi^{-1}(W_{1}^{-1}(z_{2}(x,y))))(\partial/\partial x)z_{2}(x,y)}{\varphi_{2}(\psi^{-1}(W_{1}^{-1}(z_{2}(x,y))))} \\ &\quad\geq -\sum_{i=1}^{l_{1}} \alpha_{i}'(x) \int_{\beta_{i}(y)}^{\infty} \biggl[ \int_{\alpha_{i}(x)}^{\infty} \int_{t}^{\infty}c_{i}(\xi,\eta,X,Y) \varphi_{2} \bigl(\psi^{-1} \bigl(A(\xi,\eta) \bigr) \bigr)\,d\xi \,d\eta \biggr]\,dt. \end{aligned}$$

Replace *x* with *s*, and integrating it from *x* to ∞, we get

2.29 $$ W_{2} \bigl(z_{2}(x,y) \bigr)\leq W_{2} \bigl(z_{2}(\infty,y) \bigr)+F(x,y,X,Y), $$

where

$$\begin{aligned} F(x,y,X,Y)= \sum_{i=1}^{l_{1}} \int_{\alpha_{i}(x)}^{\infty} \int_{\beta_{i}(y)}^{\infty} \biggl[ \int_{s}^{\infty} \int_{t}^{\infty}c_{i}(\xi,\eta,X,Y) \varphi_{2} \bigl(\psi^{-1} \bigl(A(\xi,\eta) \bigr) \bigr) \,d\xi \,d\eta \biggr]\,ds\,dt. \end{aligned}$$

Obviously, $F(x,y,x,y)=F(x,y)$, which is defined in (2.10). From (2.19), (2.20), (2.25), (2.26) and (2.29), we have

2.30 $$\begin{aligned} z(x,y)&\leq z_{1}(x,y)\leq W_{1}^{-1} \bigl(z_{2}(x,y) \bigr) \\ &\leq W_{1}^{-1} \bigl\{ W_{2}^{-1} \bigl\{ W_{2} \bigl[W_{1} \bigl(B(M,N)+C(M,N) \bigr)+E(X,Y) \bigr]+F(x,y,X,Y) \bigr\} \bigr\} , \\ &\quad \forall(x,y)\in[X,\infty)\times[Y,\infty). \end{aligned}$$

Since *X* and *Y* are chosen arbitrarily, we have

2.31 $$ \begin{aligned}[b] &z(x,y)\leq z_{1}(x,y)\leq W_{1}^{-1} \bigl\{ W_{2}^{-1} \bigl\{ W_{2} \bigl[W_{1} \bigl(B(M,N)+C(M,N) \bigr)+E(x,y) \bigr]+F(x,y) \bigr\} \bigr\} ,\hspace{-20pt} \\ &\quad \forall(x,y)\in[M,\infty)\times[N,\infty). \end{aligned} $$

By the definition of $C(M,N)$ and (2.19), we get

2.32 $$\begin{aligned} C(M,N)&\leq z_{1}(M,N)D(M,N) \\ &\leq W_{1}^{-1} \bigl\{ W_{2}^{-1} \bigl\{ W_{2} \bigl[W_{1} \bigl(B(M,N)+C(M,N) \bigr)+E(M,N) \bigr] \\ &\quad {}+F(M,N) \bigr\} \bigr\} D(M,N), \end{aligned}$$

or

2.33 $$ W_{2} \biggl[W_{1} \biggl(\frac{C(M,N)}{D(M,N)} \biggr) \biggr]-W_{2} \bigl[W_{1} \bigl(B(M,N)+C(M,N) \bigr)+E(M,N) \bigr]\leq F(M,N), $$

where $D(M,N)$ is defined in (2.8). By (2.5) and the hypothesis of *G*, we obtain

2.34 $$ C(M,N)\leq G^{-1} \bigl(F(M,N) \bigr). $$

Combining (2.31), (2.34) and (2.14), we get the desired result. □

### Corollary 2.1

*Let the functions* *k*, *a*, *α*, $b_{i}$, $c_{i}$, $\alpha_{i}$, $\beta_{i}$ ($i=1,2,\ldots,l_{1}$), $d_{j}$, $e_{j}$, $\alpha_{j}$, $\beta_{j}$ ($j=1,2,\ldots,l_{2}$) *and* *u* *be defined as in Theorem* 2.1, *p* *is a positive constant and* $p\geq1$. *If the function* $u(x,y)$ *satisfies the inequality*,

2.35 $$\begin{aligned} u^{p}(x,y)&\leq k(x,y)+ \int_{\alpha(x)}^{\infty}a(s,y)u^{p}(s,y)\,ds +\sum _{i=1}^{l_{1}} \int_{\alpha_{i}(x)}^{\infty} \int_{\beta_{i}(y)}^{\infty} \biggl[b_{i}(s,t,x,y)u(s,t) \\ &\quad{}+ \int_{s}^{\infty} \int_{t}^{\infty}c_{i}(\xi,\eta,x,y)\max _{\sigma\in [\xi,h\xi]} u(\sigma,\eta)\,d\xi \,d\eta \biggr]\,ds\,dt \\ &\quad {} +\sum _{j=1}^{l_{2}} \int_{\alpha_{j}(M)}^{\infty} \int_{\beta_{j}(N)}^{\infty} \biggl[d_{j}(s,t,x,y)u^{p}(s,t) \\ &\quad{}+ \int_{s}^{\infty} \int_{t}^{\infty}e_{j}(\xi,\eta,x,y)\max _{\sigma\in [\xi,h\xi]} u^{p}(\sigma,\eta))\,d\xi \,d\eta \biggr]\,ds \,dt, \quad (x,y)\in\Delta, \end{aligned}$$

*then*: (i) *if* $p>1$, *we have*

2.36 $$ \begin{aligned}[b] u(x,y)&\leq \biggl\{ \biggl\{ k(x,y)\\ &\quad {}+ \biggl[ \bigl[\widetilde{B}(M,N)+G_{1}^{-1} \bigl(\widetilde{F}(M,N) \bigr) \bigr]^{\frac{p-1}{p}} +\frac{p-1}{p} \widetilde{F}(x,y) \biggr]^{\frac{p}{p-1}} \biggr\} A(x,y) \biggr\} ^{\frac{1}{p}}, \end{aligned} $$

*where*

2.37 $$\begin{aligned}& \widetilde{B}(M,N)=\sum_{i=1}^{l_{1}} \int_{\alpha_{i}(M)}^{\infty} \int_{\beta_{i}(N)}^{\infty} \biggl[b_{i}(s,t,M,N)k^{\frac{1}{p}}(s,t)A^{\frac{1}{p}}(s,t) \\& \hphantom{ \widetilde{B}(M,N)=}{}+ \int_{s}^{\infty} \int_{t}^{\infty}c_{i}(\xi, \eta,M,N)k^{\frac{1}{p}}(\xi,\eta)A^{\frac{1}{p}}(\xi,\eta)\,d\xi \,d\eta \biggr]\,ds\,dt \\& \hphantom{ \widetilde{B}(M,N)=}{}+\sum_{j=1}^{l_{2}} \int_{\alpha_{j}(M)}^{\infty} \int_{\beta_{j}(N)}^{\infty} \biggl[d_{j}(s,t,M,N)k(s,t)A(s,t) \\& \hphantom{ \widetilde{B}(M,N)=}{}+ \int_{s}^{\infty} \int_{t}^{\infty}e_{j}(\xi,\eta,M,N)k(\xi, \eta)A(\xi,\eta)\,d\xi \,d\eta \biggr]\,ds\,dt, \end{aligned}$$

2.38 $$\begin{aligned}& \widetilde{F}(x,y)=\sum_{i=1}^{l_{1}} \int_{\alpha_{i}(x)}^{\infty} \int_{\beta_{i}(y)}^{\infty} \biggl[b_{i}(s,t,x,y)A^{\frac{1}{p}}(s,t) \\& \hphantom{\widetilde{F}(x,y)=}{}+ \int_{s}^{\infty} \int_{t}^{\infty}c_{i}(\xi, \eta,x,y)A^{\frac{1}{p}}(\xi,\eta)\,d\xi \,d\eta \biggr]\,ds\,dt, \end{aligned}$$

2.39 $$\begin{aligned}& G_{1}(u)=\frac{p}{p-1} \biggl[ \biggl(\frac{u}{D(M,N)} \biggr)^{\frac{p-1}{p}}- \bigl(\widetilde{B}(M,N)+u \bigr)^{\frac{p-1}{p}} \biggr], \end{aligned}$$

*on condition that* $G_{1}(u)$ *is a strictly increasing function on* $R_{+}$.

(ii) *If* $p=1$, *we have*

2.40 $$ u(x,y)\leq \biggl[k(x,y)+\frac{\overline{B}(M,N)\exp{(\overline{F}(x,y))}}{1-D(M,N)\exp{(\overline{F}(M,N))}} \biggr]A(x,y), $$

*where*

2.41 $$ D(M,N)\exp{ \bigl(\overline{F}(M,N) \bigr)}< 1 $$

*and*

2.42 $$\begin{aligned}& \begin{aligned}[b] \overline{B}(M,N)&=\sum_{i=1}^{l_{1}} \int_{\alpha_{i}(M)}^{\infty} \int_{\beta_{i}(N)}^{\infty} \biggl[b_{i}(s,t,M,N)k(s,t)A(s,t) \\ &\quad{}+ \int_{s}^{\infty} \int_{t}^{\infty}c_{i}(\xi,\eta,M,N)k(\xi, \eta)A(\xi,\eta)\,d\xi \,d\eta \biggr]\,ds\,dt \\ &\quad{}+\sum_{j=1}^{l_{2}} \int_{\alpha_{j}(M)}^{\infty} \int_{\beta_{j}(N)}^{\infty} \biggl[d_{j}(s,t,M,N)k(s,t)A(s,t) \\ &\quad{}+ \int_{s}^{\infty} \int_{t}^{\infty}e_{j}(\xi,\eta,M,N)k(\xi, \eta)A(\xi,\eta)\,d\xi \,d\eta \biggr]\,ds\,dt, \end{aligned} \end{aligned}$$

2.43 $$\begin{aligned}& \begin{aligned}[b] \overline{F}(x,y)&=\sum_{i=1}^{l_{1}} \int_{\alpha_{i}(x)}^{\infty} \int_{\beta_{i}(y)}^{\infty} \biggl[b_{i}(s,t,x,y)A(s,t) \\ &\quad {}+ \int_{s}^{\infty} \int_{t}^{\infty}c_{i}(\xi,\eta,x,y)A(\xi, \eta)\,d\xi \,d\eta \biggr]\,ds\,dt. \end{aligned} \end{aligned}$$

### Proof

Inequality (2.35) followed by letting $\psi(u(x,y))=u^{p}(x,y)$, $\varphi_{1}(u(x,y))=\varphi_{2}(u(x,y))=u(x,y)$ in Theorem 2.1. Then $\psi^{-1}(u(x,y))=u^{\frac{1}{p}}(x,y)$ and $(u+v)^{\frac{1}{p}}\leq u^{\frac{1}{p}}+v^{\frac{1}{p}}$, $(uv)^{\frac{1}{p}}= u^{\frac{1}{p}}v^{\frac{1}{p}}$.

If $p>1$, we have

$$\begin{aligned} &W_{1}(z)= \int_{c}^{z}\frac{du}{u^{1/p}}= \frac{p}{p-1}z^{\frac{p-1}{p}}-\frac{p}{p-1}c^{\frac{p-1}{p}}, \\ &W_{1}^{-1}(z)= \biggl(\frac{p-1}{p}z+c^{\frac{p-1}{p}} \biggr)^{\frac{p}{p-1}}. \end{aligned} $$

Applying Theorem 2.1, we can easily get (2.36).

If $p=1$, we have

$$\begin{aligned} &W_{1}(z)= \int_{c}^{z}\frac{du}{u}=\ln z-\ln c, \quad \quad W_{1}^{-1}(z)=c\exp{z}, \\ &G_{2}(u)=W_{1} \biggl(\frac{u}{D(M,N)} \biggr)-W_{1} \bigl(\overline{B}(M,N)+u \bigr)=\ln{\frac{u}{D(M,N)(\overline{B}(M,N)+u)}}. \end{aligned}$$

Obviously, $G_{2}(u)$ is a strictly increasing function on $R_{+}$, $G_{2}^{-1}(u)$ is the inverse of $G_{2}(u)$, we get

$$\begin{aligned} &G_{2}^{-1}(u)=\frac{\overline{B}(M,N)D(M,N)\exp{(u)}}{1-D(M,N)\exp{(u)}},\quad D(M,N)\exp{(u)}< 1, \end{aligned}$$

where $\overline{B}(M,N)$ is defined in (2.42). Applying Theorem 2.1, we can easily get (2.40). Details are omitted here. □

### Theorem 2.2

*Suppose that the following conditions hold*:

(i) (ii)-(iv) *of Theorem* 2.1 *are satisfied*;
(ii) $q_{i}$, $r_{i}$ *are nonnegative constants with* $p\geq q_{i}$, $p\geq r_{i}$, $i=1,2,\ldots,l_{1}$, *and* $\varepsilon_{j}$, $\delta_{j}$ *are nonnegative constants with* $p\geq\varepsilon_{j}$, $p\geq\delta_{j}$, $j=1,2,\ldots,l_{2}$.

*If* $(x,y)\in\Delta$, $u(x,y)$ *satisfies the following inequality*:

2.44 $$\begin{aligned} u^{p}(x,y)&\leq k(x,y)+ \int_{\alpha(x)}^{\infty}a(s,y)u^{p}(s,y)\,ds +\sum _{i=1}^{l_{1}} \int_{\alpha_{i}(x)}^{\infty} \int_{\beta_{i}(y)}^{\infty} \biggl[b_{i}(s,t,x,y)u^{q_{i}}(s,t) \\ &\quad{}+ \int_{s}^{\infty} \int_{t}^{\infty}c_{i}(\xi,\eta,x,y)\max _{\sigma\in [\xi,h\xi]} u^{r_{i}}(\sigma,\eta)\,d\xi \,d\eta \biggr]\,ds \,dt \\ &\quad {} +\sum_{j=1}^{l_{2}} \int_{\alpha_{j}(M)}^{\infty} \int_{\beta_{j}(N)}^{\infty} \biggl[d_{j}(s,t,x,y)u^{\varepsilon_{j}}(s,t) \\ &\quad{}+ \int_{s}^{\infty} \int_{t}^{\infty}e_{j}(\xi,\eta,x,y)\max _{\sigma\in [\xi,h\xi]} u^{\delta_{j}}(\sigma,\eta))\,d\xi \,d\eta \biggr]\,ds \,dt, \quad (x,y)\in\Delta, \end{aligned}$$

*then we have*

2.45 $$ u(x,y)\leq \biggl\{ \biggl[k(x,y)+\frac{B_{1}(M,N)}{1-D_{1}(M,N)}\exp{ \bigl(F_{1}(x,y) \bigr)} \biggr]A(x,y) \biggr\} ^{\frac{1}{p}},\quad (x,y)\in\Delta, $$

*where*

2.46 $$\begin{aligned}& \begin{aligned}[b] B_{1}(M,N)&=\sum_{i=1}^{l_{1}} \int_{\alpha_{i}(M)}^{\infty} \int_{\beta_{i}(N)}^{\infty} \biggl\{ b_{i}(s,t,M,N) A^{\frac{q_{i}}{p}}(s,t) \biggl[\frac{q_{i}}{p}K_{1}^{\frac{q_{i}-p}{p}}k(s,t)+ \frac{p-q_{i}}{p}K_{1}^{\frac{q_{i}}{p}} \biggr] \\ &\quad{}+ \int_{s}^{\infty} \int_{t}^{\infty}c_{i}(\xi,\eta,M,N) A^{\frac{r_{i}}{p}}(\xi,\eta)\\ &\quad {}\times \biggl[\frac{r_{i}}{p}K_{2}^{\frac{r_{i}-p}{p}}k( \xi,\eta)+\frac{p-r_{i}}{p}K_{2}^{\frac{r_{i}}{p}} \biggr] \,d\xi \,d \eta \biggr\} \,ds\,dt \\ &\quad{}+\sum_{j=1}^{l_{2}} \int_{\alpha_{j}(M)}^{\infty} \int_{\beta_{j}(N)}^{\infty} \biggl\{ d_{j}(s,t,M,N) A^{\frac{\varepsilon_{j}}{p}}(s,t) \biggl[\frac{\varepsilon_{j}}{p}K_{3}^{\frac{\varepsilon_{j}-p}{p}}k(s,t)+ \frac{p-\varepsilon_{j}}{p}K_{3}^{\frac{\varepsilon_{j}}{p}} \biggr]\hspace{-20pt} \\ &\quad{}+ \int_{s}^{\infty} \int_{t}^{\infty}e_{j}(\xi,\eta,M,N) A^{\frac{\delta_{j}}{p}}(\xi,\eta)\\ &\quad {}\times \biggl[\frac{\delta_{j}}{p}K_{4}^{\frac{\delta_{j}-p}{p}}k( \xi,\eta)+\frac{p-\delta_{j}}{p}K_{4}^{\frac{\delta_{j}}{p}} \biggr] \,d\xi \,d \eta \biggr\} \,ds\,dt, \end{aligned} \end{aligned}$$

2.47 $$\begin{aligned}& \begin{aligned}[b] F_{1}(x,y)&=\sum_{i=1}^{l_{1}} \int_{\alpha_{i}(x)}^{\infty} \int_{\beta_{i}(y)}^{\infty} \biggl[b_{i}(s,t,x,y)A^{\frac{q_{i}}{p}}(s,t) \frac{q_{i}}{p}K_{1}^{\frac{q_{i}-p}{p}} \\ &\quad{}+ \int_{s}^{\infty} \int_{t}^{\infty}c_{i}(\xi, \eta,x,y)A^{\frac{r_{i}}{p}}(\xi,\eta)\frac{r_{i}}{p}K_{2}^{\frac{r_{i}-p}{p}} \,d \xi \,d\eta \biggr]\,ds\,dt, \end{aligned} \end{aligned}$$

2.48 $$\begin{aligned}& \begin{aligned}[b] D_{1}(M,N)&=\sum_{j=1}^{l_{2}} \int_{\alpha_{j}(M)}^{\infty} \int_{\beta_{j}(N)}^{\infty} \biggl[d_{j}(s,t,M,N) A^{\frac{\varepsilon_{j}}{p}}(s,t)\frac{\varepsilon_{j}}{p}K_{3}^{\frac{\varepsilon_{j}-p}{p}}\exp{ \bigl(F_{1}(s,t) \bigr)} \\ &\quad{}+ \int_{s}^{\infty} \int_{t}^{\infty}e_{j}(\xi,\eta,M,N) A^{\frac{\delta_{j}}{p}}(\xi,\eta)\frac{\delta_{j}}{p}K_{4}^{\frac{\delta_{j}-p}{p}}\exp{ \bigl(F_{1}(\xi,\eta) \bigr)}\,d\xi \,d\eta \biggr]\,ds\,dt\hspace{-20pt}\\ & < 1. \end{aligned} \end{aligned}$$

### Proof

Let

2.49 $$\begin{aligned} z(x,y)&=\sum_{i=1}^{l_{1}} \int_{\alpha_{i}(x)}^{\infty} \int_{\beta_{i}(y)}^{\infty} \biggl[b_{i}(s,t,x,y)u^{q_{i}}(s,t) \\ &\quad{}+ \int_{s}^{\infty} \int_{t}^{\infty}c_{i}(\xi,\eta,x,y)\max _{\sigma\in [\xi,h\xi]} u^{r_{i}}(\sigma,\eta)\,d\xi \,d\eta \biggr]\,ds \,dt \\ &\quad{}+\sum_{j=1}^{l_{2}} \int_{\alpha_{j}(M)}^{\infty} \int_{\beta_{j}(N)}^{\infty} \biggl[d_{j}(s,t,x,y)u^{\varepsilon_{j}}(s,t) \\ &\quad{}+ \int_{s}^{\infty} \int_{t}^{\infty}e_{j}(\xi,\eta,x,y)\max _{\sigma\in [\xi,h\xi]} u^{\delta_{j}}(\sigma,\eta))\,d\xi \,d\eta \biggr]\,ds \,dt. \end{aligned}$$

Obviously, $z(x,y)$ is non-increasing in every variable. From (2.44) and (2.49), we have

2.50 $$ u^{p}(x,y)\leq k(x,y)+z(x,y)+ \int_{\alpha(x)}^{\infty}a(s,y)u^{p}(s,y) \,ds. $$

By Lemma 2.2, we obtain

2.51 $$ u^{p}(x,y)\leq \bigl[k(x,y)+z(x,y) \bigr]A(x,y),\quad(x,y)\in[M, \infty)\times[N,\infty), $$

where $A(x,y)$ is defined in (2.6). Then we get

2.52 $$ u(x,y)\leq \bigl[ \bigl(k(x,y)+z(x,y) \bigr)A(x,y) \bigr]^{\frac{1}{p}}, \quad(x,y)\in[M,\infty)\times[N,\infty). $$

By Lemma 2.3, we have

2.53 $$ \begin{gathered} u^{q_{i}}(x,y)\leq \bigl[ \bigl(k(x,y)+z(x,y) \bigr)A(x,y) \bigr]^{\frac{q_{i}}{p}} \\ \hphantom{u^{q_{i}}(x,y)}\leq A^{\frac{q_{i}}{p}}(x,y) \biggl[\frac{q_{i}}{p}K_{1}^{\frac{q_{i}-p}{p}} \bigl(k(x,y)+z(x,y) \bigr)+\frac{p-q_{i}}{p}K_{1}^{\frac{q_{i}}{p}} \biggr] \\ \hphantom{u^{q_{i}}(x,y)}=A^{\frac{q_{i}}{p}}(x,y) \biggl[\frac{q_{i}}{p}K_{1}^{\frac{q_{i}-p}{p}}k(x,y)+ \frac{p-q_{i}}{p}K_{1}^{\frac{q_{i}}{p}}+\frac{q_{i}}{p}K_{1}^{\frac{q_{i}-p}{p}}z(x,y) \biggr], \\ \max_{\xi\in[x,hx]}u^{r_{i}}(\xi,y)\leq \max _{\xi\in[x,hx]} \bigl[ \bigl(k(\xi,y)+z(\xi,y) \bigr)A(\xi,y) \bigr]^{\frac{r_{i}}{p}} \\ \hphantom{\max_{\xi\in[x,hx]}u^{r_{i}}(\xi,y)}\leq \Bigl[ \Bigl(\max_{\xi\in[x,hx]}k(\xi,y)+\max _{\xi\in[x,hx]}z(\xi,y) \Bigr)\max_{\xi\in[x,hx]}A(\xi,y) \Bigr]^{\frac{r_{i}}{p}} \\ \hphantom{\max_{\xi\in[x,hx]}u^{r_{i}}(\xi,y)}\leq A^{\frac{r_{i}}{p}}(x,y) \biggl[\frac{r_{i}}{p}K_{2}^{\frac{r_{i}-p}{p}} \bigl(k(x,y)+z(x,y) \bigr)+\frac{p-r_{i}}{p}K_{2}^{\frac{r_{i}}{p}} \biggr] \\ \hphantom{\max_{\xi\in[x,hx]}u^{r_{i}}(\xi,y)}=A^{\frac{r_{i}}{p}}(x,y) \biggl[\frac{r_{i}}{p}K_{2}^{\frac{r_{i}-p}{p}}k(x,y)+ \frac{p-r_{i}}{p}K_{2}^{\frac{r_{i}}{p}}+\frac{r_{i}}{p}K_{2}^{\frac{r_{i}-p}{p}}z(x,y) \biggr], \\ u^{\varepsilon_{j}}(x,y)\leq A^{\frac{\varepsilon_{j}}{p}}(x,y) \biggl[\frac{\varepsilon_{j}}{p}K_{3}^{\frac{\varepsilon_{j}-p}{p}}k(x,y)+ \frac{p-\varepsilon_{j}}{p}K_{3}^{\frac{\varepsilon_{j}}{p}}+\frac{\varepsilon_{j}}{p}K_{3}^{\frac{\varepsilon_{j}-p}{p}}z(x,y) \biggr], \\ \max_{\xi\in[x,hx]}u^{\delta_{j}}(\xi,y)\leq A^{\frac{\delta_{j}}{p}}(x,y) \biggl[\frac{\delta_{j}}{p}K_{4}^{\frac{\delta_{j}-p}{p}}k(x,y)+ \frac{p-\delta_{j}}{p}K_{4}^{\frac{\delta_{j}}{p}}+\frac{\delta_{j}}{p}K_{4}^{\frac{\delta_{j}-p}{p}}z(x,y) \biggr]. \end{gathered} $$

Combining (2.53) and (2.49), we have

2.54 $$\begin{aligned} z(x,y)&\leq \sum_{i=1}^{l_{1}} \int_{\alpha_{i}(x)}^{\infty} \int_{\beta_{i}(y)}^{\infty} \biggl\{ b_{i}(s,t,x,y) A^{\frac{q_{i}}{p}}(s,t) \biggl[\frac{q_{i}}{p}K_{1}^{\frac{q_{i}-p}{p}}k(s,t) \\ &\quad {} +\frac{p-q_{i}}{p}K_{1}^{\frac{q_{i}}{p}}+\frac{q_{i}}{p}K_{1}^{\frac{q_{i}-p}{p}}z(s,t) \biggr] \\ &\quad{}+ \int_{s}^{\infty} \int_{t}^{\infty}c_{i}(\xi,\eta,x,y) A^{\frac{r_{i}}{p}}(\xi,\eta) \biggl[\frac{r_{i}}{p}K_{2}^{\frac{r_{i}-p}{p}}k( \xi,\eta) \\ &\quad{}+\frac{p-r_{i}}{p}K_{2}^{\frac{r_{i}}{p}}+\frac{r_{i}}{p}K_{2}^{\frac{r_{i}-p}{p}}z( \xi,\eta) \biggr]\,d\xi \,d\eta \biggr\} \,ds\,dt \\ &\quad{}+\sum_{j=1}^{l_{2}} \int_{\alpha_{j}(M)}^{\infty} \int_{\beta_{j}(N)}^{\infty} \biggl\{ d_{j}(s,t,x,y) A^{\frac{\varepsilon_{j}}{p}}(s,t) \biggl[\frac{\varepsilon_{j}}{p}K_{3}^{\frac{\varepsilon_{j}-p}{p}}k(s,t) \\ &\quad{}+\frac{p-\varepsilon_{j}}{p}K_{3}^{\frac{\varepsilon_{j}}{p}}+\frac{\varepsilon_{j}}{p}K_{3}^{\frac{\varepsilon_{j}-p}{p}}z(s,t) \biggr] \\ &\quad{}+ \int_{s}^{\infty} \int_{t}^{\infty}e_{j}(\xi,\eta,x,y) A^{\frac{\delta_{j}}{p}}(\xi,\eta) \biggl[\frac{\delta_{j}}{p}K_{4}^{\frac{\delta_{j}-p}{p}}k( \xi,\eta) \\ &\quad{}+\frac{p-\delta_{j}}{p}K_{4}^{\frac{\delta_{j}}{p}}+\frac{\delta_{j}}{p}K_{4}^{\frac{\delta_{j}-p}{p}}z( \xi,\eta) \biggr]\,d\xi \,d\eta \biggr\} \,ds\,dt \\ &=B_{1}(x,y)+C_{1}(x,y) +\sum _{i=1}^{l_{1}} \int_{\alpha_{i}(x)}^{\infty} \int_{\beta_{i}(y)}^{\infty} \biggl[b_{i}(s,t,x,y) A^{\frac{q_{i}}{p}}(s,t)\frac{q_{i}}{p}K_{1}^{\frac{q_{i}-p}{p}}z(s,t) \\ &\quad{}+ \int_{s}^{\infty} \int_{t}^{\infty}c_{i}(\xi,\eta,x,y) A^{\frac{r_{i}}{p}}(\xi,\eta)\frac{r_{i}}{p}K_{2}^{\frac{r_{i}-p}{p}}z(\xi, \eta)\,d\xi \,d\eta \biggr]\,ds\,dt \\ &\leq B_{1}(M,N)+C_{1}(M,N) \\ &\quad {} +\sum _{i=1}^{l_{1}} \int_{\alpha_{i}(x)}^{\infty} \int_{\beta_{i}(y)}^{\infty} \biggl[b_{i}(s,t,x,y) A^{\frac{q_{i}}{p}}(s,t)\frac{q_{i}}{p}K_{1}^{\frac{q_{i}-p}{p}}z(s,t) \\ &\quad{}+ \int_{s}^{\infty} \int_{t}^{\infty}c_{i}(\xi,\eta,x,y) A^{\frac{r_{i}}{p}}(\xi,\eta)\frac{r_{i}}{p}K_{2}^{\frac{r_{i}-p}{p}}z(\xi, \eta)\,d\xi \,d\eta \biggr]\,ds\,dt, \\ & \quad(x,y)\in[M,\infty)\times[N, \infty), \end{aligned}$$

where $B_{1}(M,N)$ is defined in (2.46), $C_{1}(M,N)$ is defined as follows:

2.55 $$\begin{aligned} C_{1}(M,N)&=\sum_{j=1}^{l_{2}} \int_{\alpha_{j}(M)}^{\infty} \int_{\beta_{j}(N)}^{\infty} \biggl[d_{j}(s,t,M,N) A^{\frac{\varepsilon_{j}}{p}}(s,t)\frac{\varepsilon_{j}}{p}K_{3}^{\frac{\varepsilon_{j}-p}{p}}z(s,t) \\ &\quad{}+ \int_{s}^{\infty} \int_{t}^{\infty}e_{j}(\xi,\eta,M,N) A^{\frac{\delta_{j}}{p}}(\xi,\eta)\frac{\delta_{j}}{p}K_{4}^{\frac{\delta_{j}-p}{p}}z(\xi, \eta)\,d\xi \,d\eta \biggr]\,ds\,dt. \end{aligned}$$

$\forall X\in I_{1}$, $\forall Y\in I_{2}$, for all $(x,y)\in[X,\infty)\times[Y,\infty)$, we have

2.56 $$\begin{aligned} z(x,y)&\leq B_{1}(M,N)+C_{1}(M,N) +\sum _{i=1}^{l_{1}} \int_{\alpha_{i}(x)}^{\infty} \int_{\beta_{i}(y)}^{\infty} \biggl[b_{i}(s,t,X,Y) A^{\frac{q_{i}}{p}}(s,t)\frac{q_{i}}{p}K_{1}^{\frac{q_{i}-p}{p}}z(s,t) \\ &\quad{}+ \int_{s}^{\infty} \int_{t}^{\infty}c_{i}(\xi,\eta,X,Y) A^{\frac{r_{i}}{p}}(\xi,\eta)\frac{r_{i}}{p}K_{2}^{\frac{r_{i}-p}{p}}z(\xi, \eta)\,d\xi \,d\eta \biggr]\,ds\,dt. \end{aligned}$$

Let $z_{1}(x,y)$ denote the function on the right-hand side of (2.56), which is positive and non-increasing in each of the variables $(x,y)\in[X,\infty)\times[Y,\infty)$. From (2.56), we have

2.57 $$\begin{aligned} &z(x,y)\leq z_{1}(x,y),\quad(x,y)\in[X,\infty)\times[Y, \infty), \end{aligned}$$

2.58 $$\begin{aligned} &z_{1}(\infty,y)=B_{1}(M,N)+C_{1}(M,N). \end{aligned}$$

Differentiating $z_{1}(x,y)$ with respect to *x*, we have

2.59 $$\begin{aligned} \frac{\partial z_{1}(x,y)}{\partial x}&=-\sum_{i=1}^{l_{1}} \alpha_{i}'(x) \int_{\beta_{i}(y)}^{\infty} \biggl[b_{i} \bigl( \alpha_{i}(x),t,X,Y \bigr) A^{\frac{q_{i}}{p}} \bigl(\alpha_{i}(x),t \bigr)\frac{q_{i}}{p}K_{1}^{\frac{q_{i}-p}{p}}z \bigl( \alpha_{i}(x),t \bigr) \\ &\quad{}+ \int_{\alpha_{i}(x)}^{\infty} \int_{t}^{\infty}c_{i}(\xi,\eta,X,Y) A^{\frac{r_{i}}{p}}(\xi,\eta)\frac{r_{i}}{p}K_{2}^{\frac{r_{i}-p}{p}}z(\xi, \eta)\,d\xi \,d\eta \biggr]\,dt \\ &\geq -\sum_{i=1}^{l_{1}} \alpha_{i}'(x) \int_{\beta_{i}(y)}^{\infty} \biggl[b_{i} \bigl( \alpha_{i}(x),t,X,Y \bigr) A^{\frac{q_{i}}{p}} \bigl(\alpha_{i}(x),t \bigr)\frac{q_{i}}{p}K_{1}^{\frac{q_{i}-p}{p}}z_{1} \bigl( \alpha_{i}(x),t \bigr) \\ &\quad{}+ \int_{\alpha_{i}(x)}^{\infty} \int_{t}^{\infty}c_{i}(\xi,\eta,X,Y) A^{\frac{r_{i}}{p}}(\xi,\eta)\frac{r_{i}}{p}K_{2}^{\frac{r_{i}-p}{p}}z_{1}( \xi,\eta)\,d\xi \,d\eta \biggr]\,dt. \end{aligned}$$

Dividing both sides of (2.59) by $z_{1}(x,y)$, noticing that $z_{1}(x,y)$ is non-increasing in each variable, we have

2.60 $$\begin{aligned} \frac{(\partial/\partial x)z_{1}(x,y)}{z_{1}(x,y)}&\geq -\sum_{i=1}^{l_{1}} \alpha_{i}'(x) \int_{\beta_{i}(y)}^{\infty} \biggl[b_{i} \bigl( \alpha_{i}(x),t,X,Y \bigr) A^{\frac{q_{i}}{p}} \bigl(\alpha_{i}(x),t \bigr)\frac{q_{i}}{p}K_{1}^{\frac{q_{i}-p}{p}} \\ &\quad{}+ \int_{\alpha_{i}(x)}^{\infty} \int_{t}^{\infty}c_{i}(\xi,\eta,X,Y) A^{\frac{r_{i}}{p}}(\xi,\eta)\frac{r_{i}}{p}K_{2}^{\frac{r_{i}-p}{p}}\,d\xi \,d \eta \biggr]\,dt, \\ &\quad (x,y)\in[X,\infty)\times[Y,\infty). \end{aligned}$$

Replace *x* with *s*, and integrate it from *x* to ∞, we get

2.61 $$ z_{1}(x,y)\leq z_{1}(\infty,y)\exp{ \bigl(F_{1}(x,y,X,Y) \bigr)},\quad(x,y)\in[X,\infty)\times[Y,\infty), $$

where

2.62 $$\begin{aligned} F_{1}(x,y,X,Y)&=\sum_{i=1}^{l_{1}} \int_{\alpha_{i}(x)}^{\infty} \int_{\beta_{i}(y)}^{\infty} \biggl[b_{i}(s,t,X,Y) A^{\frac{q_{i}}{p}}(s,t)\frac{q_{i}}{p}K_{1}^{\frac{q_{i}-p}{p}} \\ &\quad{}+ \int_{s}^{\infty} \int_{t}^{\infty}c_{i}(\xi,\eta,X,Y) A^{\frac{r_{i}}{p}}(\xi,\eta)\frac{r_{i}}{p}K_{2}^{\frac{r_{i}-p}{p}}\,d\xi \,d \eta \biggr]\,ds\,dt. \end{aligned}$$

It is obvious that $F_{1}(x,y,x,y)=F_{1}(x,y)$, which is defined in (2.47). From (2.57), (2.58) and (2.61), we get

2.63 $$ z(x,y)\leq \bigl[B_{1}(M,N)+C_{1}(M,N) \bigr]\exp{ \bigl(F_{1}(x,y,X,Y) \bigr)},\quad(x,y)\in[X,\infty)\times[Y, \infty). $$

Due to the fact that *X*, *Y* are chosen arbitrarily, we have

2.64 $$ z(x,y)\leq \bigl[B_{1}(M,N)+C_{1}(M,N) \bigr]\exp{ \bigl(F_{1}(x,y) \bigr)},\quad(x,y)\in[M,\infty)\times[N, \infty). $$

By the definition of $C_{1}(M,N)$, we have

2.65 $$\begin{aligned} B_{1}(M,N)+C_{1}(M,N)&\leq B_{1}(M,N)+\sum _{j=1}^{l_{2}} \int_{\alpha_{j}(M)}^{\infty} \int_{\beta_{j}(N)}^{\infty} \biggl[d_{j}(s,t,M,N) A^{\frac{\varepsilon_{j}}{p}}(s,t)\frac{\varepsilon_{j}}{p}K_{3}^{\frac{\varepsilon_{j}-p}{p}} \\ &\quad{}\times \bigl[B_{1}(M,N)+C_{1}(M,N) \bigr]\exp{ \bigl(F_{1}(s,t) \bigr)} \\ &\quad{}+ \int_{s}^{\infty} \int_{t}^{\infty}e_{j}(\xi,\eta,M,N) A^{\frac{\delta_{j}}{p}}(\xi,\eta)\frac{\delta_{j}}{p}K_{4}^{\frac{\delta_{j}-p}{p}} \\ &\quad{}\times \bigl[B_{1}(M,N)+C_{1}(M,N) \bigr]\exp{ \bigl(F_{1}(\xi,\eta) \bigr)}\,d\xi \,d\eta \biggr]\,ds\,dt \\ &\leq B_{1}(M,N)+ \bigl[B_{1}(M,N)+C_{1}(M,N) \bigr]D_{1}(M,N), \end{aligned}$$

where $D_{1}(M,N)$ is defined in (2.48). Then, according to $D_{1}(M,N)<1$, we have

2.66 $$ B_{1}(M,N)+C_{1}(M,N)\leq \frac{B_{1}(M,N)}{1-D_{1}(M,N)}. $$

From (2.64) and (2.66), we get

2.67 $$ z(x,y)\leq \frac{B_{1}(M,N)}{1-D_{1}(M,N)}\exp{ \bigl(F_{1}(x,y) \bigr)}, \quad(x,y) \in[M,\infty)\times[N,\infty). $$

Combining (2.52) and (2.67), we obtain the desired result. □

### Remark 2.1

If $q_{i}=r_{i}=1$ ($i=1,2,\ldots,l_{1}$), $\varepsilon_{j}=\delta_{j}=p$ ($j=1,2,\ldots,l_{2}$), the inequality (2.44) becomes (2.35), but the proof of Theorem 2.2 is different from that of Corollary 2.1.

### Corollary 2.2

*Let* *k*, *a*, *α*, $\alpha_{i}$, $\beta_{i}$, $b_{i}$, $c_{i}$ ($i=1,2,\ldots,l_{1}$), $\alpha_{j}$, $\beta_{j}$, $d_{j}$, $e_{j}$ ($i=1,2,\ldots,l_{2}$) *be defined as in Theorem* 2.1, *then* *q*, *r* *are nonnegative constants with* $0\leq q\leq 2$, $0\leq r\leq 2$. *For* $(x,y)\in\Delta$, $u(x,y)$ *satisfies the following inequality*:

2.68 $$\begin{aligned} u^{2}(x,y)&\leq k(x,y)+ \int_{\alpha(x)}^{\infty}a(s,y)u^{2}(s,y)\,ds \\ &\quad {} +\sum _{i=1}^{l_{1}} \int_{\alpha_{i}(x)}^{\infty} \int_{\beta_{i}(y)}^{\infty} \biggl[b_{i}(s,t,x,y)u^{q}(s,t) \\ &\quad{}+ \int_{s}^{\infty} \int_{t}^{\infty}c_{i}(\xi,\eta,x,y)\max _{\sigma\in [\xi,h\xi]} u^{r}(\sigma,\eta)\,d\xi \,d\eta \biggr]\,ds \,dt \\ &\quad {} +\sum_{j=1}^{l_{2}} \int_{\alpha_{j}(M)}^{\infty} \int_{\beta_{j}(N)}^{\infty} \biggl[d_{j}(s,t,x,y)u(s,t) \\ &\quad{}+ \int_{s}^{\infty} \int_{t}^{\infty}e_{j}(\xi,\eta,x,y)\max _{\sigma\in [\xi,h\xi]} u(\sigma,\eta))\,d\xi \,d\eta \biggr]\,ds\,dt,\quad (x,y) \in \Delta, \end{aligned}$$

*then we have*

2.69 $$ u(x,y)\leq \biggl\{ \biggl[k(x,y)+\frac{B_{2}(M,N)}{1-D_{2}(M,N)}\exp{ \bigl(F_{2}(x,y) \bigr)} \biggr]A(x,y) \biggr\} ^{\frac{1}{2}}, $$

*where*

2.70 $$\begin{aligned}& \begin{aligned}[b] F_{2}(x,y)&=\sum_{i=1}^{l_{1}} \int_{\alpha_{i}(x)}^{\infty} \int_{\beta_{i}(y)}^{\infty} \biggl[b_{i}(s,t,x,y)A^{\frac{q}{2}}(s,t) \frac{q}{2}K_{1}^{\frac{q-2}{2}} \\ &\quad{}+ \int_{s}^{\infty} \int_{t}^{\infty}c_{i}(\xi, \eta,x,y)A^{\frac{r}{2}}(\xi,\eta)\frac{r}{2}K_{2}^{\frac{r-2}{2}} \,d \xi \,d\eta \biggr]\,ds\,dt, \end{aligned} \end{aligned}$$

2.71 $$\begin{aligned}& \begin{aligned}[b] & B_{2}(M,N)\\ &\quad =\sum_{i=1}^{l_{1}} \int_{\alpha_{i}(M)}^{\infty} \int_{\beta_{i}(N)}^{\infty} \biggl\{ b_{i}(s,t,M,N) A^{\frac{q}{2}}(s,t) \biggl[\frac{q}{2}K_{1}^{\frac{q-2}{2}}k(s,t)+ \frac{2-q}{2}K_{1}^{\frac{q}{2}} \biggr] \\ &\qquad{}+ \int_{s}^{\infty} \int_{t}^{\infty}c_{i}(\xi,\eta,M,N) A^{\frac{r}{2}}(\xi,\eta) \biggl[\frac{r}{2}K_{2}^{\frac{r-2}{2}}k( \xi,\eta)+\frac{2-r}{2}K_{2}^{\frac{r}{2}} \biggr] \,d\xi \,d \eta \biggr\} \,ds\,dt \\ &\qquad{}+\sum_{j=1}^{l_{2}} \int_{\alpha_{j}(M)}^{\infty} \int_{\beta_{j}(N)}^{\infty} \biggl\{ d_{j}(s,t,M,N) A^{\frac{1}{2}}(s,t) \biggl[\frac{1}{2}K_{3}^{-\frac{1}{2}}k(s,t)+ \frac{1}{2}K_{3}^{\frac{1}{2}} \biggr] \\ &\qquad{}+ \int_{s}^{\infty} \int_{t}^{\infty}e_{j}(\xi,\eta,M,N) A^{\frac{1}{2}}(\xi,\eta) \biggl[\frac{1}{2}K_{4}^{-\frac{1}{2}}k( \xi,\eta)+\frac{1}{2}K_{4}^{\frac{1}{2}} \biggr] \,d\xi \,d \eta \biggr\} \,ds\,dt, \end{aligned} \end{aligned}$$

2.72 $$\begin{aligned}& \begin{aligned}[b] D_{2}(M,N)&=\sum_{j=1}^{l_{2}} \int_{\alpha_{j}(M)}^{\infty} \int_{\beta_{j}(N)}^{\infty} \biggl[d_{j}(s,t,M,N) A^{\frac{1}{2}}(s,t)\frac{1}{2}K_{3}^{-\frac{1}{2}}\exp{ \bigl(F_{2}(s,t) \bigr)} \\ &\quad{}+ \int_{s}^{\infty} \int_{t}^{\infty}e_{j}(\xi,\eta,M,N) A^{\frac{1}{2}}(\xi,\eta)\frac{1}{2}K_{4}^{-\frac{1}{2}}\exp{ \bigl(F_{2}(\xi,\eta) \bigr)}\,d\xi \,d\eta \biggr]\,ds\,dt\\ &< 1. \end{aligned} \end{aligned}$$

### Proof

Inequality (2.68) follows by inequality (2.44) with $p=2$, $q_{i}=q$, $r_{i}=r$ ($i=1,2,\ldots,l_{1}$), $\varepsilon_{j}=\delta_{j}=1$ ($j=1,2,\ldots,l_{2}$). Then, applying Theorem 2.2, we can easily get (2.69). Details are omitted here. □

### Remark 2.2

As one can see, the established results above mainly deal with Volterra-Fredholm type integral inequalities with maxima in two variables. And they are different from the results presented in [14, 21, 23]. In Theorem 2.1, in the case of one variable, if we take $k(x,y)=k$, $a(x,y)=0$, $l_{1}=l_{2}=1$, $b_{1}(s,t,x,y)=d_{1}(s,t,x,y)=h_{1}(s)$, $c_{1}(\xi,\eta,x,y)=e_{1}(\xi,\eta,x,y)=h_{2}(\xi)$, $\psi(u)=\varphi_{1}(u)$, $\psi(u)=\varphi_{2}(u)$ in the second iterated integral, orderly, we will get the inequality that is similar to inequality (1.5). If the above conditions are satisfied in two dimensions and $\varphi_{2}(\max_{\sigma\in[\xi,h\xi]}u(\sigma,\eta))=\varphi_{2}(u(\xi,\eta))$, we get analogs of the inequality (1.4). And if we take $l_{1}=2$, $l_{2}=0$, $b_{i}(s,t,x,y)=f_{i}(s,t)$, $c_{i}(\xi,\eta,x,y)=0$ in Theorem 2.1, inequality (2.1) reduces to (1.3).

## Applications in the integral equation

In this section, we apply our results in Theorem 2.1 and Theorem 2.2 to study the retarded Volterra-Fredholm type integral equations with maxima in two variables. Some results on the boundedness of their solutions are presented, which demonstrate that our results can be used to investigate the qualitative properties of solutions of some integral equations.

### Example

We consider the retarded Volterra-Fredholm type integral equation of the form

3.1 $$\begin{aligned} \psi \bigl(v(x,y) \bigr)&=g_{1}(x,y)+ \int_{x}^{\infty}g_{2}(s,y)\psi \bigl(v \bigl(s+\rho(s),y \bigr) \bigr)\,ds \\ &\quad{}+\sum_{i=1}^{l_{1}} \int_{x}^{\infty} \int_{y}^{\infty}F_{1i} \biggl(s,t,x,y,v \bigl(s+\rho_{i}(s),t+\gamma_{i}(t) \bigr), \\ &\quad \int_{s}^{\infty} \int_{t}^{\infty}F_{2i} \Bigl(s,t,x,y,\max _{\sigma\in[\xi+\rho_{i}(\xi),h(\xi+\rho_{i}(\xi))]}v \bigl(\sigma,\eta+\gamma_{i}(\eta) \bigr) \Bigr) \,d\xi \,d\eta \biggr)\,ds\,dt \\ &\quad{}+\sum_{j=1}^{l_{2}} \int_{M}^{\infty} \int_{N}^{\infty}G_{1j} \biggl(s,t,x,y,v \bigl(s+\rho_{j}(s),t+\gamma_{j}(t) \bigr), \\ &\quad \int_{s}^{\infty} \int_{t}^{\infty}G_{2j} \Bigl(s,t,x,y,\max _{\sigma\in[\xi+\rho_{j}(\xi),h(\xi+\rho_{j}(\xi))]}v \bigl(\sigma,\eta+\gamma_{j}(\eta) \bigr) \Bigr) \,d\xi \,d\eta \biggr)\,ds\,dt, \\ &\quad (x,y)\in \Delta. \end{aligned}$$

Suppose that the following conditions hold:

(i) $g_{1}(x,y)$, $g_{2}(x,y)$, $v(x,y)\in C(\Delta,R)$;
(ii) $x+\rho(x)$, $x+\rho_{i}(x)$, $x+\rho_{j}(x)\in C^{1}(I_{1},I_{1})$ and $y+\gamma_{i}(y)$, $y+\gamma_{j}(y)\in C^{1}(I_{2},I_{2})$ are strictly increasing with
  $$\begin{gathered} \rho(M)=\rho_{i}(M)=\rho_{j}(M)=0,\qquad \gamma_{i}(N)=\gamma_{j}(N)=0, \\ \rho(x)\geq 0,\qquad \rho_{i}(x)\geq 0,\qquad \rho_{j}(x) \geq 0\quad \mbox{for }x\geq M, \\ \gamma_{i}(y)\geq 0,\qquad \gamma_{j}(y)\geq 0\quad \mbox{for }y\geq N, \\ \rho'(x)>-1,\qquad \rho_{i}'(x)>-1,\qquad \rho_{j}'(x)>-1,\\ \gamma_{i}'(y)>-1, \qquad \gamma_{j}'(y)>-1\quad (i=1,2, \ldots,l_{1}; j=1,2,\ldots,l_{2}); \end{gathered} $$
(iii) $F_{1i}, G_{1j}\in C(\Delta^{2}\times R^{2},R)$, $F_{2i}, G_{2j}\in C(\Delta^{2}\times R,R)$ ($i=1,2,\ldots,l_{1}$; $j=1,2,\ldots,l_{2}$).

Let $\alpha(x)=x+\rho(x)$, $\alpha_{i}(x)=x+\rho_{i}(x)$, $\alpha_{j}(x)=x+\rho_{j}(x)$, $\beta_{i}(y)=y+\gamma_{i}(y)$, $\beta_{j}(y)=y+\gamma_{j}(y)$. Then *α*, $\alpha_{i}$, $\alpha_{j}$, $\beta_{i}$, $\beta_{j}$ satisfy the condition (iv) of Theorem 2.1.

### Theorem 3.1

*In Eq*. (3.1), *suppose that the following conditions hold*:

3.2 $$ \begin{aligned} &\psi \bigl(v(x,y) \bigr)=v(x,y), \qquad \bigl\vert g_{1}(x,y) \bigr\vert \leq k(x,y), \qquad \bigl\vert g_{2}(x,y) \bigr\vert \leq a(x,y), \\ & \bigl\vert F_{1i}(s,t,x,y,u,v) \bigr\vert \leq b_{i}(s,t,x,y)\varphi_{1} \bigl( \vert u \vert \bigr)+ \vert v \vert , \\ & \bigl\vert F_{2i}(s,t,x,y,u) \bigr\vert \leq c_{i}(s,t,x,y)\varphi_{2} \bigl( \vert u \vert \bigr), \quad i=1,2,\ldots,l_{1}, \\ & \bigl\vert G_{1j}(s,t,x,y,u,v) \bigr\vert \leq d_{j}(s,t,x,y) \vert u \vert + \vert v \vert , \\ & \bigl\vert G_{2j}(s,t,x,y,u) \bigr\vert \leq e_{j}(s,t,x,y) \vert u \vert ,\quad j=1,2,\ldots,l_{2}, \end{aligned} $$

*where* *k*, *a*, $b_{i}$, $c_{i}$, $d_{j}$, $e_{j}$, $\varphi_{1}$, $\varphi_{2}$ *are defined in Theorem* 2.1. *Assume that the function* $G_{3}(u)=W_{2} (W_{1}(\frac{u}{D_{3}(M,N)}) )-W_{2} (W_{1}(B_{3}(M,N)+u)+E_{3}(M,N) )$ *is increasing*. *Then we have the following estimate*:

3.3 $$\begin{aligned} \bigl\vert v(x,y) \bigr\vert &\leq \bigl[k(x,y)+W_{1}^{-1} \bigl\{ W_{2}^{-1} \bigl\{ W_{2} \bigl[W_{1} \bigl(B_{3}(M,N)+G_{3}^{-1} \bigl(F_{3}(M,N) \bigr) \bigr) \\ &\quad{}+E_{3}(x,y) \bigr]+F_{3}(x,y) \bigr\} \bigr\} \bigr]A_{1}(x,y), \quad (x,y)\in\Delta, \end{aligned}$$

*where*

3.4 $$\begin{aligned}& A_{1}(x,y)=\exp{ \biggl( \int_{\alpha(x)}^{\infty}M_{1}a \bigl( \alpha^{-1}(s),y \bigr)\,ds \biggr)}, \end{aligned}$$

3.5 $$\begin{aligned}& \begin{aligned}[b] &B_{3}(M,N)\\ &\quad =\sum _{i=1}^{l_{1}} \int_{\alpha_{i}(M)}^{\infty} \int_{\beta_{i}(N)}^{\infty} M_{1i}M_{2i} \biggl[b_{i} \bigl(\alpha_{i}^{-1}(s), \beta_{i}^{-1}(t),M,N \bigr)\varphi_{1} \bigl(k(s,t)A_{1}(s,t) \bigr) \\ &\qquad{}+ \int_{s}^{\infty} \int_{t}^{\infty}M_{1i}M_{2i}c_{i} \bigl(\alpha_{i}^{-1}(\xi),\beta_{i}^{-1}( \eta),M,N \bigr)\varphi_{2} \bigl(k(\xi,\eta)A_{1}(\xi, \eta) \bigr) \,d\xi \,d\eta \biggr]\,ds\,dt \\ &\qquad{}+\sum_{j=1}^{l_{2}} \int_{\alpha_{j}(M)}^{\infty} \int_{\beta_{j}(N)}^{\infty} M_{1j}M_{2j} \biggl[d_{j} \bigl(\alpha_{j}^{-1}(s), \beta_{j}^{-1}(t),M,N \bigr)k(s,t)A_{1}(s,t) \\ &\qquad{}+ \int_{s}^{\infty} \int_{t}^{\infty}M_{1j}M_{2j}e_{j} \bigl(\alpha_{j}^{-1}(\xi),\beta_{j}^{-1}( \eta),M,N \bigr)k(\xi,\eta)A_{1}(\xi,\eta)\,d\xi \,d\eta \biggr]\,ds \,dt, \end{aligned} \end{aligned}$$

3.6 $$\begin{aligned}& \begin{aligned}[b] D_{3}(M,N)&=\sum _{j=1}^{l_{2}} \int_{\alpha_{j}(M)}^{\infty} \int_{\beta_{j}(N)}^{\infty} M_{1j}M_{2j} \biggl[d_{j} \bigl(\alpha_{j}^{-1}(s), \beta_{j}^{-1}(t),M,N \bigr)A_{1}(s,t) \\ &\quad{}+ \int_{s}^{\infty} \int_{t}^{\infty}M_{1j}M_{2j}e_{j} \bigl(\alpha_{j}^{-1}(\xi),\beta_{j}^{-1}( \eta),M,N \bigr)A_{1}(\xi,\eta)\,d\xi \,d\eta \biggr]\,ds\,dt, \end{aligned} \end{aligned}$$

3.7 $$\begin{aligned}& E_{3}(M,N)=\sum_{i=1}^{l_{1}} \int_{\alpha_{i}(M)}^{\infty} \int_{\beta_{i}(N)}^{\infty} M_{1i}M_{2i}b_{i} \bigl(\alpha_{i}^{-1}(s),\beta_{i}^{-1}(t),M,N \bigr)\varphi_{1} \bigl(A_{1}(s,t) \bigr)\,ds \,dt, \end{aligned}$$

3.8 $$\begin{aligned}& \begin{aligned}[b] F_{3}(x,y)&=\sum_{i=1}^{l_{1}} \int_{\alpha_{i}(x)}^{\infty} \int_{\beta_{i}(y)}^{\infty} M_{1i}^{2}M_{2i}^{2}\\ &\quad {}\times \biggl[ \int_{s}^{\infty} \int_{t}^{\infty}c_{i} \bigl( \alpha_{i}^{-1}(\xi),\beta_{i}^{-1}( \eta),x,y \bigr)\varphi_{2}(A_{1}(\xi,\eta) \,d\xi \,d\eta \biggr]\,ds\,dt, \end{aligned} \end{aligned}$$

3.9 $$\begin{aligned}& \begin{gathered} M_{1}=\max_{x\in I_{1}} \frac{1}{\alpha'(\alpha^{-1}(x))}< \infty, \\ M_{1i}=\max_{x\in I_{1}} \frac{1}{\alpha_{i}'(\alpha_{i}^{-1}(x))}< \infty,\qquad M_{2i}=\max_{y\in I_{2}} \frac{1}{\beta_{i}'(\beta_{i}^{-1}(y))}< \infty,\quad i=1,2,\ldots,l_{1}; \\ M_{1j}=\max_{x\in I_{1}} \frac{1}{\alpha_{j}'(\alpha_{j}^{-1}(x))}< \infty, \qquad M_{2j}=\max_{y\in I_{2}} \frac{1}{\beta_{j}'(\beta_{j}^{-1}(y))}< \infty, \quad j=1,2,\ldots,l_{2}; \end{gathered} \end{aligned}$$

$W_{1}$, $W_{2}$ *are defined in Theorem* 2.1.

### Proof

By applying the conditions (3.2) to (3.1), we have

3.10 $$\begin{aligned} \bigl\vert v(x,y) \bigr\vert &\leq k(x,y)+ \int_{x}^{\infty}a(s,y)\cdot \bigl\vert v \bigl(s+ \rho(s),y \bigr) \bigr\vert \,ds \\ &\quad{}+\sum_{i=1}^{l_{1}} \int_{x}^{\infty} \int_{y}^{\infty} \biggl[b_{i}(s,t,x,y) \varphi_{1} \bigl( \bigl\vert v \bigl(s+\rho_{i}(s),t+ \gamma_{i}(t) \bigr) \bigr\vert \bigr) \\ &\quad{}+ \int_{s}^{\infty} \int_{t}^{\infty}c_{i}(\xi,\eta,x,y) \varphi_{2} \Bigl( \Bigl\vert \max_{\sigma\in[\xi+\rho_{i}(\xi),h(\xi+\rho_{i}(\xi))]}v \bigl( \sigma,\eta+\gamma_{i}(\eta) \bigr) \Bigr\vert \Bigr) \,d\xi \,d\eta \biggr]\,ds\,dt \\ &\quad{}+\sum_{j=1}^{l_{2}} \int_{M}^{\infty} \int_{N}^{\infty} \biggl[d_{j}(s,t,x,y) \bigl\vert v \bigl(s+\rho_{j}(s),t+\gamma_{j}(t) \bigr) \bigr\vert \\ &\quad{}+ \int_{s}^{\infty} \int_{t}^{\infty}e_{j}(\xi,\eta,x,y) \Bigl\vert \max_{\sigma\in[\xi+\rho_{j}(\xi),h(\xi+\rho_{j}(\xi))]}v \bigl(\sigma,\eta+\gamma_{j}( \eta) \bigr) \Bigr\vert \,d\xi \,d\eta \biggr]\,ds\,dt \\ &\leq k(x,y)+ \int_{\alpha(x)}^{\infty}a \bigl(\alpha^{-1}(s),y \bigr) \bigl\vert v(s,y) \bigr\vert \frac{1}{\alpha'(\alpha^{-1}(s))}\,ds \\ &\quad{}+\sum_{i=1}^{l_{1}} \int_{\alpha_{i}(x)}^{\infty} \int_{\beta_{i}(y)}^{\infty} \biggl[b_{i} \bigl( \alpha_{i}^{-1}(s),\beta_{i}^{-1}(t),x,y \bigr)\varphi_{1} \bigl( \bigl\vert v(s,t) \bigr\vert \bigr) \\ &\quad {} + \int_{s}^{\infty} \int_{t}^{\infty}c_{i} \bigl( \alpha_{i}^{-1}(\xi),\beta_{i}^{-1}( \eta),x,y \bigr)\varphi_{2} \Bigl(\max_{\sigma\in[\xi,h\xi]} \bigl\vert v( \sigma,\eta) \bigr\vert \Bigr) \\ &\quad{}\times \frac{1}{\alpha_{i}'(\alpha_{i}^{-1}(\xi))}\frac{1}{\beta_{i}'(\beta_{i}^{-1}(\eta))} \,d\xi \,d\eta \biggr] \frac{1}{\alpha_{i}'(\alpha_{i}^{-1}(s))}\frac{1}{\beta_{i}'(\beta_{i}^{-1}(t))}\,ds\,dt \\ &\quad{}+\sum_{j=1}^{l_{2}} \int_{\alpha_{j}(M)}^{\infty} \int_{\beta_{j}(N)}^{\infty} \biggl[d_{j} \bigl( \alpha_{j}^{-1}(s),\beta_{j}^{-1}(t),x,y \bigr) \bigl\vert v(s,t) \bigr\vert \\ &\quad {} + \int_{s}^{\infty} \int_{t}^{\infty}e_{j} \bigl( \alpha_{j}^{-1}(\xi),\beta_{j}^{-1}( \eta),x,y \bigr) \\ &\quad{}\times\max_{\sigma\in[\xi,h\xi]} \bigl\vert v(\sigma,\eta) \bigr\vert \frac{1}{\alpha_{j}'(\alpha_{j}^{-1}(\xi))}\frac{1}{\beta_{j}'(\beta_{j}^{-1}(\eta))}\,d\xi \,d\eta \biggr] \frac{1}{\alpha_{j}'(\alpha_{j}^{-1}(s))} \frac{1}{\beta_{j}'(\beta_{j}^{-1}(t))}\,ds\,dt \\ &\leq k(x,y)+ \int_{\alpha(x)}^{\infty}M_{1}a \bigl( \alpha^{-1}(s),y \bigr) \bigl\vert v(s,y) \bigr\vert \,ds \\ &\quad{}+\sum_{i=1}^{l_{1}} \int_{\alpha_{i}(x)}^{\infty} \int_{\beta_{i}(y)}^{\infty} \biggl[M_{1i}M_{2i}b_{i} \bigl(\alpha_{i}^{-1}(s),\beta_{i}^{-1}(t),x,y \bigr)\varphi_{1} \bigl( \bigl\vert v(s,t) \bigr\vert \bigr) \\ &\quad{}+ \int_{s}^{\infty} \int_{t}^{\infty}M_{1i}^{2}M_{2i}^{2} c_{i} \bigl(\alpha_{i}^{-1}(\xi), \beta_{i}^{-1}(\eta),x,y \bigr)\varphi_{2} \Bigl( \max_{\sigma\in[\xi,h\xi]} \bigl\vert v(\sigma,\eta) \bigr\vert \Bigr)\,d\xi \,d \eta \biggr]\,ds\,dt \\ &\quad{}+\sum_{j=1}^{l_{2}} \int_{\alpha_{j}(M)}^{\infty} \int_{\beta_{j}(N)}^{\infty} \biggl[M_{1j}M_{2j}d_{j} \bigl(\alpha_{j}^{-1}(s),\beta_{j}^{-1}(t),x,y \bigr) \bigl\vert v(s,t) \bigr\vert \\ &\quad{}+ \int_{s}^{\infty} \int_{t}^{\infty}M_{1j}^{2}M_{2j}^{2} e_{j} \bigl(\alpha_{j}^{-1}(\xi), \beta_{j}^{-1}(\eta),x,y \bigr) \\ &\quad {}\times\max_{\sigma\in[\xi,h\xi]} \bigl\vert v(\sigma,\eta) \bigr\vert \,d\xi \,d\eta \biggr]\,ds \,dt, \end{aligned}$$

for $(x,y)\in\Delta$, where $M_{1}$, $M_{1i}$, $M_{2i}$ ($i=1,2,\ldots,l_{1}$), $M_{1j}$, $M_{2j}$ ($j=1,2,\ldots,l_{2}$) are defined in (3.9). Applying the results of Theorem 2.1 to (3.10) with $\psi(u)=u$, $a(s,y)=M_{1}a(\alpha^{-1}(s),y)$, $b_{i}(s,t,x,y)=M_{1i}M_{2i}b_{i}(\alpha_{i}^{-1}(s),\beta_{i}^{-1}(t),x,y)$, $c_{i}(\xi,\eta,x,y)=M_{1i}^{2}M_{2i}^{2}c_{i}(\alpha_{i}^{-1}(\xi),\beta_{i}^{-1}(\eta),x,y)$, $d_{j}(s,t,x,y)=M_{1j}M_{2j}d_{j}(\alpha_{j}^{-1}(s),\beta_{j}^{-1}(t),x,y)$, $e_{j}(\xi,\eta,x,y)=M_{1j}^{2}M_{2j}^{2}e_{j}(\alpha_{j}^{-1}(\xi),\beta_{j}^{-1}(\eta),x,y)$, we obtain the desired estimation (3.3). □

### Theorem 3.2

*In equation* (3.1), *suppose that the following conditions hold*:

3.11 $$ \begin{aligned} &\psi \bigl(v(x,y) \bigr)=v^{p}(x,y), \qquad \bigl\vert g_{1}(x,y) \bigr\vert \leq k(x,y), \qquad \bigl\vert g_{2}(x,y) \bigr\vert \leq a(x,y), \\ & \bigl\vert F_{1i}(s,t,x,y,u,v) \bigr\vert \leq b_{i}(s,t,x,y) \vert u \vert ^{q_{i}}+ \vert v \vert , \\ & \bigl\vert F_{2i}(s,t,x,y,u) \bigr\vert \leq c_{i}(s,t,x,y) \vert u \vert ^{r_{i}},\quad i=1,2, \ldots,l_{1}, \\ & \bigl\vert G_{1j}(s,t,x,y,u,v) \bigr\vert \leq d_{j}(s,t,x,y) \vert u \vert ^{\varepsilon_{j}}+ \vert v \vert , \\ & \bigl\vert G_{2j}(s,t,x,y,u) \bigr\vert \leq e_{j}(s,t,x,y) \vert u \vert ^{\delta_{j}},\quad j=1,2, \ldots,l_{2}, \end{aligned} $$

*where* *p*, $q_{i}$, $r_{i}$, $\varepsilon_{j}$, $\delta_{j}$, $b_{i}$, $c_{i}$, $d_{j}$, $e_{j}$ ($i=1,2,\ldots,l_{1}$; $j=1,2,\ldots,l_{2}$) *are defined as in Theorem* 2.2. *Then we have the following estimate*:

3.12 $$ \bigl\vert v(x,y) \bigr\vert \leq \biggl\{ \biggl[k(x,y)+\frac{B_{4}(M,N)}{1-D_{4}(M,N)} \exp{ \bigl(F_{4}(x,y) \bigr)} \biggr]A_{1}(x,y) \biggr\} ^{\frac{1}{p}}, $$

*where*

3.13 $$\begin{aligned}& \begin{aligned}[b] B_{4}(M,N)&=\sum _{i=1}^{l_{1}} \int_{\alpha_{i}(M)}^{\infty} \int_{\beta_{i}(N)}^{\infty}M_{1i}M_{2i} \biggl\{ b_{i} \bigl(\alpha_{i}^{-1}(s), \beta_{i}^{-1}(t),M,N \bigr) A_{1}^{\frac{q_{i}}{p}}(s,t) \\ &\quad{}\times \biggl[\frac{q_{i}}{p}K_{1}^{\frac{q_{i}-p}{p}}k(s,t)+ \frac{p-q_{i}}{p}K_{1}^{\frac{q_{i}}{p}} \biggr]\\ &\quad {} + \int_{s}^{\infty} \int_{t}^{\infty}M_{1i}M_{2i}c_{i} \bigl(\alpha_{i}^{-1}(\xi),\beta_{i}^{-1}( \eta),M,N \bigr) \\ &\quad{}\times A_{1}^{\frac{r_{i}}{p}}(\xi,\eta) \biggl[ \frac{r_{i}}{p}K_{2}^{\frac{r_{i}-p}{p}}k( \xi,\eta)+ \frac{p-r_{i}}{p}K_{2}^{\frac{r_{i}}{p}} \biggr] \,d\xi \,d\eta \biggr\} \,ds\,dt \\ &\quad{}+\sum_{j=1}^{l_{2}} \int_{\alpha_{j}(M)}^{\infty} \int_{\beta_{j}(N)}^{\infty}M_{1j}M_{2j} \biggl\{ d_{j} \bigl(\alpha_{j}^{-1}(s), \beta_{j}^{-1}(t),M,N \bigr) A_{1}^{\frac{\varepsilon_{j}}{p}}(s,t) \\ &\quad{}\times \biggl[\frac{\varepsilon_{j}}{p}K_{3}^{\frac{\varepsilon_{j}-p}{p}}k(s,t)+ \frac{p-\varepsilon_{j}}{p}K_{3}^{\frac{\varepsilon_{j}}{p}} \biggr]\\ &\quad {} + \int_{s}^{\infty} \int_{t}^{\infty}M_{1j}M_{2j}e_{j} \bigl(\alpha_{j}^{-1}(\xi),\beta_{j}^{-1}( \eta),M,N \bigr) \\ &\quad{}\times A_{1}^{\frac{\delta_{j}}{p}}(\xi,\eta) \biggl[ \frac{\delta_{j}}{p}K_{4}^{\frac{\delta_{j}-p}{p}}k( \xi,\eta) + \frac{p-\delta_{j}}{p}K_{4}^{\frac{\delta_{j}}{p}} \biggr]\,d\xi \,d\eta \biggr\} \,ds\,dt, \end{aligned} \end{aligned}$$

3.14 $$\begin{aligned}& \begin{aligned}[b] D_{4}(M,N)&=\sum _{j=1}^{l_{2}} \int_{\alpha_{j}(M)}^{\infty} \int_{\beta_{j}(N)}^{\infty}M_{1j}M_{2j}\\ &\quad {}\times \biggl[d_{j} \bigl(\alpha_{j}^{-1}(s), \beta_{j}^{-1}(t),M,N \bigr) A_{1}^{\frac{\varepsilon_{j}}{p}}(s,t) \frac{\varepsilon_{j}}{p}K_{3}^{\frac{\varepsilon_{j}-p}{p}}\exp{ \bigl(F_{4}(s,t) \bigr)} \\ &\quad{}+ \int_{s}^{\infty} \int_{t}^{\infty}M_{1j}M_{2j}e_{j} \bigl(\alpha_{j}^{-1}(\xi),\beta_{j}^{-1}( \eta),M,N \bigr)\\ &\quad {}\times A_{1}^{\frac{\delta_{j}}{p}}(\xi,\eta)\frac{\delta_{j}}{p}K_{4}^{\frac{\delta_{j}-p}{p}} \exp{ \bigl(F_{4}(\xi,\eta) \bigr)}\,d\xi \,d\eta \biggr]\,ds\,dt \\ &< 1, \end{aligned} \end{aligned}$$

3.15 $$\begin{aligned}& \begin{aligned}[b] F_{4}(x,y)&=\sum _{i=1}^{l_{1}} \int_{\alpha_{i}(x)}^{\infty} \int_{\beta_{i}(y)}^{\infty}M_{1i}M_{2i} \biggl[b_{i} \bigl(\alpha_{i}^{-1}(s), \beta_{i}^{-1}(t),x,y \bigr) A_{1}^{\frac{q_{i}}{p}}(s,t) \frac{q_{i}}{p}K_{1}^{\frac{q_{i}-p}{p}} \\ &\quad{}+ \int_{s}^{\infty} \int_{t}^{\infty}M_{1i}M_{2i}c_{i} \bigl(\alpha_{i}^{-1}(\xi),\beta_{i}^{-1}( \eta),x,y \bigr)\\ &\quad {}\times A_{1}^{\frac{r_{i}}{p}}(\xi,\eta)\frac{r_{i}}{p}K_{2}^{\frac{r_{i}-p}{p}} \,d \xi \,d\eta \biggr]\,ds\,dt. \end{aligned} \end{aligned}$$

### Proof

Applying the conditions of (3.11) to (3.1), we have

3.16 $$\begin{aligned} \bigl\vert v(x,y) \bigr\vert ^{p}&\leq k(x,y)+ \int_{x}^{\infty}a(s,y) \bigl\vert v \bigl(s+\rho(s),y \bigr) \bigr\vert ^{p}\,ds \\ &\quad{}+\sum_{i=1}^{l_{1}} \int_{x}^{\infty} \int_{y}^{\infty} \biggl[b_{i}(s,t,x,y) \bigl\vert v \bigl(s+\rho_{i}(s),t+\gamma_{i}(t) \bigr) \bigr\vert ^{q_{i}} \\ &\quad{}+ \int_{s}^{\infty} \int_{t}^{\infty}c_{i}(\xi,\eta,x,y) \Bigl\vert \max_{\sigma\in[\xi+\rho_{i}(\xi),h(\xi+\rho_{i}(\xi))]}v \bigl(\sigma,\eta+\gamma_{i}( \eta) \bigr) \Bigr\vert ^{r_{i}} \,d\xi \,d\eta \biggr]\,ds\,dt \\ &\quad{}+\sum_{j=1}^{l_{2}} \int_{M}^{\infty} \int_{N}^{\infty} \biggl[d_{j}(s,t,x,y) \bigl\vert v \bigl(s+\rho_{j}(s),t+\gamma_{j}(t) \bigr) \bigr\vert ^{\varepsilon_{j}} \\ &\quad{}+ \int_{s}^{\infty} \int_{t}^{\infty}e_{j}(\xi,\eta,x,y) \Bigl\vert \max_{\sigma\in[\xi+\rho_{j}(\xi),h(\xi+\rho_{j}(\xi))]}v \bigl(\sigma,\eta+\gamma_{j}( \eta) \bigr) \Bigr\vert ^{\delta_{j}} \,d\xi \,d\eta \biggr]\,ds\,dt \\ &\leq k(x,y)+ \int_{\alpha(x)}^{\infty}a \bigl(\alpha^{-1}(s),y \bigr) \bigl\vert v(s,y) \bigr\vert ^{p}\frac{1}{\alpha'(\alpha^{-1}(s))}\,ds \\ &\quad{}+\sum_{i=1}^{l_{1}} \int_{\alpha_{i}(x)}^{\infty} \int_{\beta_{i}(y)}^{\infty} \biggl[b_{i} \bigl( \alpha_{i}^{-1}(s),\beta_{i}^{-1}(t),x,y \bigr) \bigl\vert v(s,t) \bigr\vert ^{q_{i}} \\ &\quad {}+ \int_{s}^{\infty} \int_{t}^{\infty} c_{i} \bigl( \alpha_{i}^{-1}(\xi),\beta_{i}^{-1}( \eta),x,y \bigr) \\ &\quad{}\times\max_{\sigma\in[\xi,h\xi]} \bigl\vert v(\sigma,\eta) \bigr\vert ^{r_{i}} \frac{1}{\alpha_{i}'(\alpha_{i}^{-1}(\xi))}\frac{1}{\beta_{i}'(\beta_{i}^{-1}(\eta))}\,d\xi \,d\eta \biggr] \frac{1}{\alpha_{i}'(\alpha_{i}^{-1}(s))}\frac{1}{\beta_{i}'(\beta_{i}^{-1}(t))}\,ds\,dt \\ &\quad{}+\sum_{j=1}^{l_{2}} \int_{\alpha_{j}(M)}^{\infty} \int_{\beta_{j}(N)}^{\infty} \biggl[d_{j} \bigl( \alpha_{j}^{-1}(s),\beta_{j}^{-1}(t),x,y \bigr) \bigl\vert v(s,t) \bigr\vert ^{\varepsilon_{j}} \\ &\quad {} + \int_{s}^{\infty} \int_{t}^{\infty}e_{j} \bigl( \alpha_{j}^{-1}(\xi),\beta_{j}^{-1}( \eta),x,y \bigr) \\ &\quad{}\times\max_{\sigma\in[\xi,h\xi]} \bigl\vert v(\sigma,\eta) \bigr\vert ^{\delta_{j}} \frac{1}{\alpha_{j}'(\alpha_{j}^{-1}(\xi))}\frac{1}{\beta_{j}'(\beta_{j}^{-1}(\eta))}\,d\xi \,d\eta \biggr] \\ &\quad {}\times \frac{1}{\alpha_{j}'(\alpha_{j}^{-1}(s))}\frac{1}{\beta_{j}'(\beta_{j}^{-1}(t))}\,ds\,dt \\ &\leq k(x,y)+ \int_{\alpha(x)}^{\infty}M_{1}a \bigl( \alpha^{-1}(s),y \bigr) \bigl\vert v(s,y) \bigr\vert ^{p} \,ds \\ &\quad{}+\sum_{i=1}^{l_{1}} \int_{\alpha_{i}(x)}^{\infty} \int_{\beta_{i}(y)}^{\infty} \biggl[M_{1i}M_{2i}b_{i} \bigl(\alpha_{i}^{-1}(s),\beta_{i}^{-1}(t),x,y \bigr) \bigl\vert v(s,t) \bigr\vert ^{q_{i}} \\ &\quad{}+ \int_{s}^{\infty} \int_{t}^{\infty}M_{1i}^{2}M_{2i}^{2} c_{i} \bigl(\alpha_{i}^{-1}(\xi), \beta_{i}^{-1}(\eta),x,y \bigr)\max_{\sigma\in[\xi,h\xi]} \bigl\vert v(\sigma,\eta) \bigr\vert ^{r_{i}}\,d\xi \,d\eta \biggr]\,ds \,dt \\ &\quad{}+\sum_{j=1}^{l_{2}} \int_{\alpha_{j}(M)}^{\infty} \int_{\beta_{j}(N)}^{\infty} \biggl[M_{1j}M_{2j}d_{j} \bigl(\alpha_{j}^{-1}(s),\beta_{j}^{-1}(t),x,y \bigr) \bigl\vert v(s,t) \bigr\vert ^{\varepsilon_{j}} \\ &\quad{}+ \int_{s}^{\infty} \int_{t}^{\infty}M_{1j}^{2}M_{2j}^{2} e_{j} \bigl(\alpha_{j}^{-1}(\xi), \beta_{j}^{-1}(\eta),x,y \bigr) \\ &\quad {}\times\max_{\sigma\in[\xi,h\xi]} \bigl\vert v(\sigma,\eta) \bigr\vert ^{\delta_{j}}\,d\xi \,d\eta \biggr]\,ds \,dt, \end{aligned}$$

for $(x,y)\in\Delta$, where $M_{1}$, $M_{1i}$, $M_{2i}$ ($i=1,2,\ldots,l_{1}$), $M_{1j}$, $M_{2j}$ ($j=1,2,\ldots,l_{2}$) are defined in (3.9). Applying the results of Theorem 2.2 to (3.16) with $a(s,y)=M_{1}a(\alpha^{-1}(s),y)$, $b_{i}(s,t,x,y)=M_{1i}M_{2i}b_{i}(\alpha_{i}^{-1}(s),\beta_{i}^{-1}(t),x,y)$, $c_{i}(\xi,\eta,x,y)=M_{1i}^{2}M_{2i}^{2}c_{i}(\alpha_{i}^{-1}(\xi),\beta_{i}^{-1}(\eta),x,y)$, $d_{j}(s,t,x,y)=M_{1j}M_{2j}d_{j}(\alpha_{j}^{-1}(s),\beta_{j}^{-1}(t),x,y)$, $e_{j}(\xi,\eta,x,y)=M_{1j}^{2}M_{2j}^{2}e_{j}(\alpha_{j}^{-1}(\xi),\beta_{j}^{-1}(\eta),x,y)$, we obtain the desired estimation (3.12). □

## Conclusion

In this paper, we established several new retarded nonlinear Volterra-Fredholm type integral inequalities with maxima in two independent variables in Theorem 2.1 and Theorem 2.2, and gave their specific cases in Corollary 2.1 and Corollary 2.2, respectively, which can be used in the analysis of the qualitative properties to solutions of integral equations with maxima. In Theorem 3.1 and Theorem 3.2, we also presented the applications to research the boundedness of solutions of retarded nonlinear Volterra-Fredholm type integral equations.

Using our method, one can further study the integral inequality with more dimensions.
